# Stabilization of Functional Recombinant Cannabinoid Receptor CB_2_ in Detergent Micelles and Lipid Bilayers

**DOI:** 10.1371/journal.pone.0046290

**Published:** 2012-10-03

**Authors:** Krishna Vukoti, Tomohiro Kimura, Laura Macke, Klaus Gawrisch, Alexei Yeliseev

**Affiliations:** National Institute on Alcohol Abuse and Alcoholism, National Institutes of Health, Bethesda, Maryland, United States of America; University of Cambridge, United Kingdom

## Abstract

Elucidation of the molecular mechanisms of activation of G protein-coupled receptors (GPCRs) is among the most challenging tasks for modern membrane biology. For studies by high resolution analytical methods, these integral membrane receptors have to be expressed in large quantities, solubilized from cell membranes and purified in detergent micelles, which may result in a severe destabilization and a loss of function. Here, we report insights into differential effects of detergents, lipids and cannabinoid ligands on stability of the recombinant cannabinoid receptor CB_2_, and provide guidelines for preparation and handling of the fully functional receptor suitable for a wide array of downstream applications. While we previously described the expression in *Escherichia coli*, purification and liposome-reconstitution of multi-milligram quantities of CB_2_, here we report an efficient stabilization of the recombinant receptor in micelles - crucial for functional and structural characterization. The effects of detergents, lipids and specific ligands on structural stability of CB_2_ were assessed by studying activation of G proteins by the purified receptor reconstituted into liposomes. Functional structure of the ligand binding pocket of the receptor was confirmed by binding of ^2^H-labeled ligand measured by solid-state NMR. We demonstrate that a concerted action of an anionic cholesterol derivative, cholesteryl hemisuccinate (CHS) and high affinity cannabinoid ligands CP-55,940 or SR-144,528 are required for efficient stabilization of the functional fold of CB_2_ in dodecyl maltoside (DDM)/CHAPS detergent solutions. Similar to CHS, the negatively charged phospholipids with the serine headgroup (PS) exerted significant stabilizing effects in micelles while uncharged phospholipids were not effective. The purified CB_2_ reconstituted into lipid bilayers retained functionality for up to several weeks enabling high resolution structural studies of this GPCR at physiologically relevant conditions.

## Introduction

Heptahelical G protein-coupled receptors (GPCRs) are integral membrane proteins involved in a wide range of physiological processes including sensory transduction and cell-to-cell communication [1]. The cannabinoid receptor CB_2_ is an attractive target for the development of drugs for management of pain, inflammation, osteoporosis, inhibition of growth of malignant gliomas and tumors of immune origin, and treatment of immunological disorders such as multiple sclerosis [2], [3], [4]. High resolution structural studies will provide critical insights into the molecular mechanisms of ligand binding and activation of CB_2_, and may guide the rational design of novel, highly specific pharmaceuticals.

Although GPCRs represent as much as 50% of pharmaceutical drug targets currently under development, the progress with structural studies has been relatively slow, in part due to the difficulties in obtaining large quantities of sufficiently pure, homogenous and functional protein. With the notable exception of rhodopsin, GPCRs are naturally expressed at low levels, and heterologous production is currently the only technically feasible way to prepare these proteins [5], [6].

In addition to the availability of large quantities of purified receptor, structural methods require that the protein is sufficiently stable over extended periods of time. While solubilization in detergents is needed for extraction of GPCRs from cell membranes and chromatographic purification, the preservation of the structural integrity of receptors in micelles is a notoriously difficult task. Unlike dark-adapted bovine rhodopsin which exhibits significant stability in detergent solutions, many GPCRs, when removed from membranes and exposed to detergents, lose their functional fold in a matter of minutes and, therefore, require additional efficient stabilization [7], [8]. The relatively few successful attempts to preserve the functional structure of purified GPCRs relied on a careful selection of mild solubilizing detergents as well as on supplementation of buffers used for purification with stabilizers such as lipids and ligands, adjustment of ionic strength and glycerol content. Yet, no general methodology for an efficient stabilization of GPCRs has been developed yet, and their low stability remains a major bottleneck for structural biology [9].

A recently introduced approach to stabilization by site-directed mutagenesis [10], [11] as well as by replacement of the large intracellular loop 3 and truncation of flexible N- and C-terminal domains succeeded in obtaining well-diffracting crystals of several class A GPCRs [12], [13], [14]. However, such modifications alter the wild type structure and are known to affect the functional properties of receptors significantly [10], [11], [15], [16], [17], [18]. Therefore, rather than performing an extensive modification of the structure of CB_2_ with poorly predictable functional consequences, in this study we explored the stabilization potential of carefully selected detergents, lipids and high affinity ligands. The minimal alteration to the native amino acid sequence of CB_2_ used in this work included addition of the small affinity tags at the N- and C-terminus of the protein [19] that, as we demonstrated, do not affect function of the receptor as determined by G protein activation and ligand-binding tests [20].

We focus on developing applications of spectroscopic techniques, in particular nuclear magnetic resonance (NMR) to studies of the full length, structurally unperturbed CB_2_ in lipid bilayers [5], [6], [19], [21], [22]. Emphasis is on studies by the solid-state NMR on purified CB_2_ reconstituted at a high protein-to-lipid ratio into lipid bilayers of a defined composition. While the high-density homogenous reconstitution of CB_2_ into liposomes was discussed in a recent publication [19], the present work addresses stabilization of CB_2_ during its expression, solubilization in detergent micelles, and chromatographic purification, with a goal of maximizing yield of fully functional receptor, in micelles as well as in lipid bilayers.

Several earlier attempts to express the recombinant CB_2_ for structural investigations failed to produce pure and fully functional receptor [23], [24], [25], [26], [27] **.** Reasons for the poor recovery of functional receptor vary and depend on the approach used to expression and purification of this protein. As we demonstrated earlier, an expression of CB_2_ in *E. coli* as a fusion with maltose binding protein leads to production of a fully functional receptor located in the bacterial plasma membrane [5]. The fusion is inserted into membranes in a “N-terminus out” orientation [28]. The membrane preparation of CB_2_ can be stored at −80°C for several years without noticeable loss of the receptor’s functionality. Therefore, the significant loss of activity of the purified CB_2_ that was reported in some of our previous publications can be attributed to severe destabilization of the protein upon its solubilization in detergent micelles and subsequent chromatographic purification. Thus it was essential to find efficient means for stabilization of the receptor in detergent solutions. This required testing of a large number of conditions that affect the functional structure of CB_2_. Our strategy relied on a development of robust and quantitative screening methods.

The strong partitioning of the hydrophobic cannabinoids into detergent micelles or the lipid matrix of liposomes prevents accurate measurement of the fraction of ligand-binding-competent CB_2_ (B_max_) [20]. Furthermore, as shown in the present study, the functional receptor has to be stabilized in micelles by an excess of cannabinoid ligand which complicates conventional radioligand-binding studies. Thus an alternative way to analyze the content of functional receptor was required to counter the problems of quantification of the radioligand-bound receptor. In this work, functional activity of CB_2_ was assessed on liposome-reconstituted receptor by measurement of rates of activation of cognate G protein as well as by ^2^H-MAS NMR using the deuterated ligand CP-55,940-d_6_.

Compared to conventional radioligand binding, the rates of nucleotide exchange on G protein activated by the agonist-bound receptor provides a more stringent as well as more comprehensive way to assess CB_2_ function. It reports not only on the ligand binding competence but also on the ability of the receptor to undergo physiologically relevant conformational changes leading to activation of cognate G proteins. The subunits of G proteins used in this assay are heterologously expressed and purified following published procedures [29], [30], and their correct posttranslational modifications, adequate purity and functional activity ensured. The reaction conditions have been optimized such that the individual subunits of G proteins were provided at concentrations significantly higher than those of the receptor to maintain linear rates of accumulation of Gα_i1_ bound to γ-^35^S-GTP, a non-hydrolizable analog of GTP [20] and to ensure that they accurately represent the fraction of the ligand-binding competent and functional protein. Furthermore, these proteoliposomes model a physiologically relevant environment enabling studies of structure and function of CB_2_ as well as its interaction with the lipid matrix.

### Experimental Strategy

**Figure 1:**
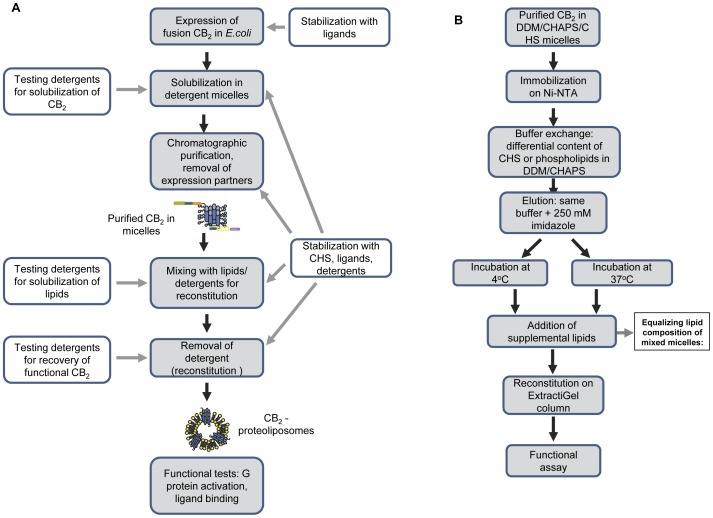
Summary of experimental strategy. A, Testing stabilizing effects of detergents, ligands and lipids. •Efficient solubilization of the fusion CB_2_ protein from *E. coli* membranes. Over 40 different detergents and mixtures of detergents were compared for their efficiency in solubilizing fusion CB_2_ from membranes, and the detergent mixture that performed best was selected for a routine receptor purification protocol. •Optimization of the liposome-reconstitution procedure. A screening of lipid-solubilizing detergents was performed with a goal of maximizing the yield of functionally reconstituted receptor. •Application of the G protein activation test to the analysis of the structural stability of CB_2_ in micelles following its reconstitution into liposomes. •Screening for stabilizers for CB_2_ and characterization of stability of the purified receptor. (i)Stabilization of CB_2_ in micelles by CHS, ligands and phospholipids. (ii)Ligand binding studies by solid-state NMR. (iii)Characterization of stability of CB_2_ in lipid bilayers. B, Comparison of stabilizing effects of two lipids at two different temperatures.

The main objective of this study has been development of procedures for efficient stabilization of the recombinant CB_2_ in detergent micelles and improvement of yield of purified, functional protein. The general outline of the experiments is presented in Fig. 1, A. The influence of detergents, cholesteryl hemisuccinate (CHS) and ligands is tested at various stages of protein preparation. The effects of stabilizers are assessed after reconstitution of the receptor into liposomes, by studying G protein activation. Whenever possible, this was done by reconstitution of the receptor into lipid bilayers of equal composition to avoid an influence from lipid composition on G protein activation rates as reported earlier [19].

To assess the effect of lipids on stability of purified CB_2_ in micelles, CHS was replaced with lipids whose stabilizing effect was to be studied (Fig. 1, B). Since these lipids end up in the reconstituted membrane and affect the activation behavior of CB_2_ [19], it is important to discern their stabilizing effects in micelles from their influence on activity of CB_2_ in lipid bilayers. This was achieved by performing a pair-wise comparison of stabilizing lipids in micelles, separating each sample into two aliquots and subjecting one of the aliquots to elevated temperature while keeping the other one at 4°C. Upon completion of this temperature treatment, the calculated amount of supplemental lipids was added to each set of samples, such as to equalize the lipid composition of micelles and, consequently, the resulting proteoliposomes. This enabled comparison of the stabilizing effects of lipids in micelles while normalizing for their possible influence on G protein activation in lipid bilayers. For a few select cases, CB_2_ integrity was evaluated by ligand binding using the deuterated ligand CP-55,940-d_6_ and ^2^H-MAS NMR.

## Results

### Solubilization of CB_2_ from *E. coli* Membranes

The efficiency of 40 non-ionic and zwitterionic detergents for solubilization of the fusion CB_2_-130 [20] from *E. coli* membranes was tested. Solubilization efficiency was analyzed by semi-quantitative Western blotting and results are summarized in Table S1. The data confirm that n-dodecyl-ß-D-maltopyranoside (DDM) in (1%) and 3-[(3-cholamidopropyl)dimethylammonio]-1-propanesulfonate (CHAPS) (0.5%) with and without supplementation with 0.1% CHS as used earlier [5], [20] are most efficient in solubilizing CB_2_. Since this particular combination of detergents was also effective in preservation of functional activity, it was used, unless otherwise noted in all subsequent experiments for extraction of the fusion CB_2_ from membranes.

### Optimization of Conditions for Functional Reconstitution

**Figure 2:**
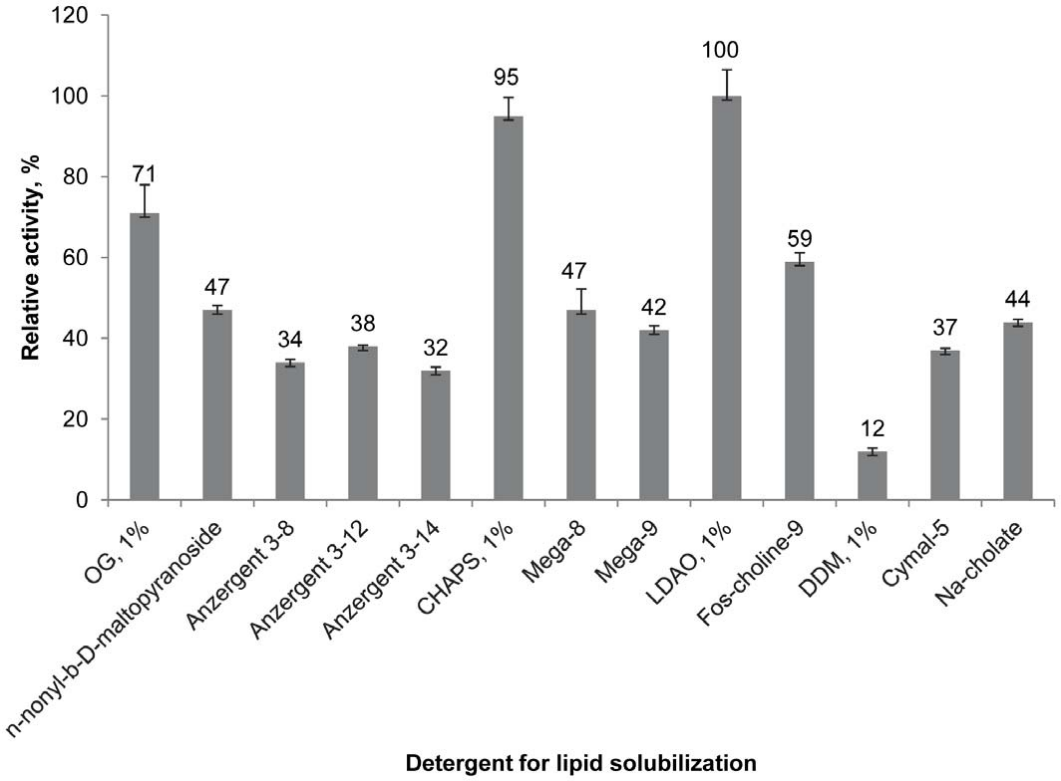
Recovery of functional CB_2_ in proteoliposomes prepared from various dominant detergents. Activity is presented as % of the maximal activity in the series (1% LDAO). The results shown represent data ± S.D. (error bars) of duplicate determinations from single representative experiments (out of three independently performed experiments, n = 3)

For functional studies not just differences in relative protein activity matter, but also the lipid/protein ratio, the homogeneity of proteoliposome fraction, and residual detergent content. These data have been reported in our previous paper [19]. For reconstitution of CB_2_, significant quantities of detergents need to be added to achieve the desired lipid/protein molar ratio in the range of 1∶100–1,000. CB_2_ was introduced into the reconstitution procedure at a concentration of 1–2 mg/mL n in buffer containing CHAPS, DDM and CHS. We examined effects of added detergents, octyl-β-D-glucopyranoside (OG), n-nonyl-β-D-maltopyranoside, n-octyl-N,N-dimethyl-3-ammonio-1-propanesulfonate/N,N-Dimethyl-N-(3-sulfopropyl)-1-octaminium hydroxide (Anzergent 3–8), Anzergent 3–12 and Anzergent 3–14), CHAPS, lauryldimethylamine-oxide (LDAO), octanoyl-N-methylglucamide (Mega 8) and nonanoyl-N-methylglucamide (Mega 9), n-nonylphosphocholine (Fos-choline 9), DDM, 5-cyclohexyl-1-pentyl-β-D-maltoside (Cymal-5) and Na-cholate (see Fig. 2). Reconstitution was performed by chromatography on detergent-absorbing resin.

A typical reconstitution procedure for 100 µg of CB_2_ on a 1.5 ml ExtractiGel column recovered 75–80% of receptor in the form of proteoliposomes. The particles were unilamellar with a mean diameter of about 120–200 nm, while the protein-to-lipid ratio typically varied between 1∶500 and 1∶600, and the content of residual detergents was ≤1 mol% as described earlier [19].

Briefly, the receptor was purified by affinity chromatography as described [20], eluted from the StrepTactin column in a “triple detergent” (TD) buffer in the final step of chromatographic purification and concentrated on a mini-spin concentrating device. This typically resulted in an increase of the protein concentration to ∼1.5–2 mg/mL accompanied by an increase in concentrations of detergents and CHS: CHAPS - to 2.5% w/v, DDM – to 0.5% w/v, CHS - to 0.5% w/v.

The optimization was performed as follows. The CB_2_ in a DDM:CHAPS:CHS detergent solution was mixed with lipids 1-palmitoyl–2-oleoyl-sn-glycero-3-phosphocholine (POPC) and 1-palmitoyl-2-oleoyl-sn-glycero-3-phospho-L-serine (POPS) (4∶1, mol/mol) dissolved in the dominant detergent to be tested. The reconstitution was performed by passing the mixture through the column of ExtractiGel resin as described, and the functional activity of CB_2_ was determined by the G protein activation assay as described in Materials and Methods. As shown in Fig. 2, the yield of the functional receptor was the highest when either CHAPS (0.5–1% (w/v)) or LDAO (0.5–1%) was the dominant detergent. Therefore, in all subsequent reconstitution experiments one of these detergents was used for preparation of the mixed micelles.

### Functional Activity of CB_2_

#### G protein activation assay

Structural integrity of the recombinant CB_2_ was assessed by measuring its functional activity by *in vitro* G protein activation [5], [20] and (for several select samples) by ^2^H- ligand-binding by solid-state NMR [19].

The rates of G protein activation can be significantly affected by a variety of conditions including ions, detergents, physical properties and chemical composition of proteoliposome particles, topology of the receptor in the bilayers, just to name a few. Therefore, the experiments were designed to normalize for possible effects from such influences. Furthermore, in addition to the G protein activation test, the ^2^H- ligand-binding assay was performed on several selected samples to relate the measured fraction of ligand binding-competent receptor to the G protein activation data.

**Figure 3:**
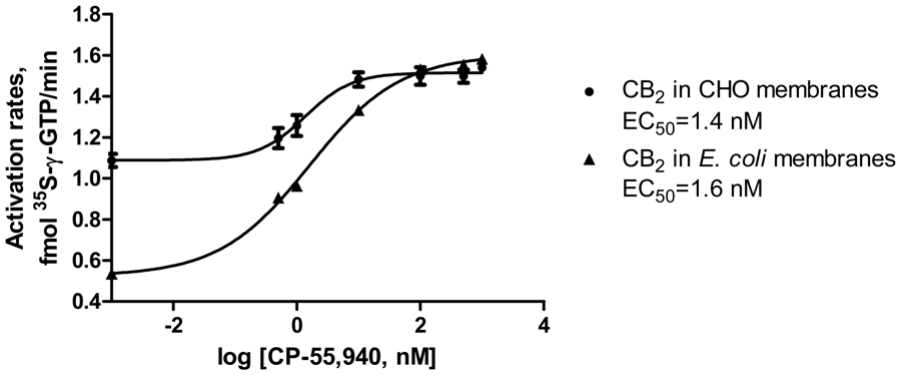
Activation of G proteins by recombinant CB_2_ in *E. coli* membranes expressing CB_2_-130 and in CHO membranes expressing CB_2_. 4 ng of CB_2_ was used in the assay and the figure depicts data ± S.D. (error bars) of duplicate determinations from representative experiments (n = 3).

The *E. coli* membranes expressing CB_2_-130 [20], devoid of endogenous G proteins, were used as a reference standard (see Materials and Methods). Since the presence of expression partners on the recombinant fusion CB_2_-130 may affect the activation rates of G proteins, we compared the performance of the fusion CB_2_ expressed in *E. coli* with activity of a membrane preparation of the native receptor expressed in CHO cells. The EC_50_ for the agonist CP-55,940 was ∼1.3 nM, identical for both membrane preparations, suggesting that the presence of the MBP- and polyhistidine tags in CB_2_-130 did not significantly affect the activation of the receptor and its interaction with G proteins (Fig. 3). Furthermore, the removal of fusion partners by treatment with the TEV protease did not alter the activation rates of G proteins on CB_2_ in membranes (results not shown).

### Effect of Residual Detergents on Activation of G Proteins

A series of co- and post-translational modification (myristoylation, palmitoylation) of heterotrimeric G proteins increases their hydrophobicity and facilitates interaction with membrane-localized GPCRs [31], [32]. To ensure an adequate solubility of several subtypes of G proteins, use of low concentrations of nonionic detergents is advisable [33]. On the other hand, detergents may affect interaction between G proteins and the receptor and influence the rate of GDP to GTP exchange [34], [35], [36]. The detergent-dependent inhibition of the G protein activation in CB_2_ membranes was assessed in the following experiments.

As is shown in Fig. S1, components of the TD buffer, at concentrations of 0.5% w/v (CHAPS) and 0.1% (DDM), respectively, completely inhibit the nucleotide exchange on G_αi1_. Ten-fold dilution of these detergents (0.05% CHAPS and 0.01% DDM) resulted in only slight (∼10%) inhibition, and lower concentration (0.005% CHAPS and 0.001% DDM) did not have any inhibitory effect.

The reaction appears to be somewhat more tolerant to the presence of octyl glycoside (OG): at concentrations of 0.05% or lower this detergent did not exhibit any negative effects. On the other hand, LDAO at the concentration 0.05% inhibited the reaction completely, and at 0.02% - by 20%. Therefore, the content of detergents in proteoliposome preparations was routinely analyzed to ensure that they were present at non-inhibitory levels. Typically, the reconstitution of CB_2_ on an absorbing resin reduces the concentration of residual detergents to below 1 mol % of lipids [19]. Furthermore, at least two dilutions of proteoliposomes were tested to determine the specific activity of the receptor, to account for possible inhibitory effects of impurities introduced with the proteoliposomes.

The above results were taken into consideration when assessing stabilizing the effects of detergents, ligand and lipids by measuring activation of G protein by CB_2_ reconstituted into proteoliposomes.

### Stabilization of CB_2_ in Micelles

The structural stability of CB_2_ was assessed by measuring the rates of G protein activation upon reconstitution of the receptor into proteoliposomes as described in Materials and Methods. To study the effects of stabilizing additives (CHS, lipids and ligands) these compounds were introduced at various stages of the expression and purification procedure as indicated in the text.

#### Stabilization by CHS

The stabilization of recombinant GPCRs in micelles by cholesteryl hemisuccinate (CHS) has been described previously [5], [7], [12], [37], [38]. As we reported earlier, CHS was added at 0.1% (w/v) to buffers used for solubilization and chromatographic purification of CB_2_ [6], [20]. The reconstitution of CB_2_ from the mixed DDM/CHAPS/CHS micelles reduces the content of detergents to below detection levels (≤1 mol%), while CHS is almost quantitatively inserted into lipid bilayers. At a protein-to-lipid ratio of ∼1∶500 the content of CHS in proteoliposomes can reach 25% (mol) of total lipids [19]. Thus, to study CB_2_ in lipid bilayers of defined composition it is essential to control the content of CHS in mixed micelles prior to reconstitution.

To test the effect of CHS on stability of CB_2_ in micelles, the biomass of *E. coli* expressing fusion CB_2_-130 was divided into two equal portions, and the recombinant protein solubilized and purified in buffers either with or without added 0.1% (w/v) CHS. Upon completion of the chromatographic purification (duration ∼ 48 hours) and proteolytic removal of fusion partners, the CB_2_ purified in the presence of CHS was mixed with lipids POPC:POPS (4∶1), and reconstituted into proteoliposomes as described in Materials and Methods. The protein sample purified in the absence of CHS was mixed with POPC:POPS:CHS (56∶14:30 w/w/w), and reconstituted using the column-absorbent procedure, such that the content of CHS, POPC and POPS in the proteoliposome preparations of proteins isolated either with or without CHS was essentially identical. Therefore, the equalized lipid composition of proteoliposomes simplified the subsequent analysis of the differences in activation rates of G proteins between these two samples, and highlighted the contribution of CHS to stabilization of CB_2_ in micellar state.

The CB_2_ isolated in the presence of CHS exhibited a robust activation of G proteins upon treatment with the high affinity agonist CP-55,940 (specific rates were 8 times higher than the background rates from spontaneous nucleotide exchange on Gα_i1_). At the same time, the receptor purified in the absence of CHS did not show any measurable activity (Fig. S2). Since both proteoliposome preparations were of identical lipid composition and protein-to-lipid ratio, these results suggest that CHS is critical to maintain the functional structure of CB_2_ in detergent micelles.

We further optimized the concentration of CHS required for efficient stabilization. The receptor was purified in a buffer containing 0.1% CHS, immobilized onto the Ni-NTA resin via the C-terminal poly-histidine tag, and the buffer was replaced with a new one, containing CHS in concentrations ranging from 0 to 0.2% (w/v). The receptor was then eluted from the resin, supplemented with a mixture of lipids POPC:POPS (4∶1 mol/mol) and reconstituted into proteoliposomes as described in Materials and Methods. The entire procedure of detergent exchange and reconstitution into liposomes took ∼3 hours.

**Figure 4:**
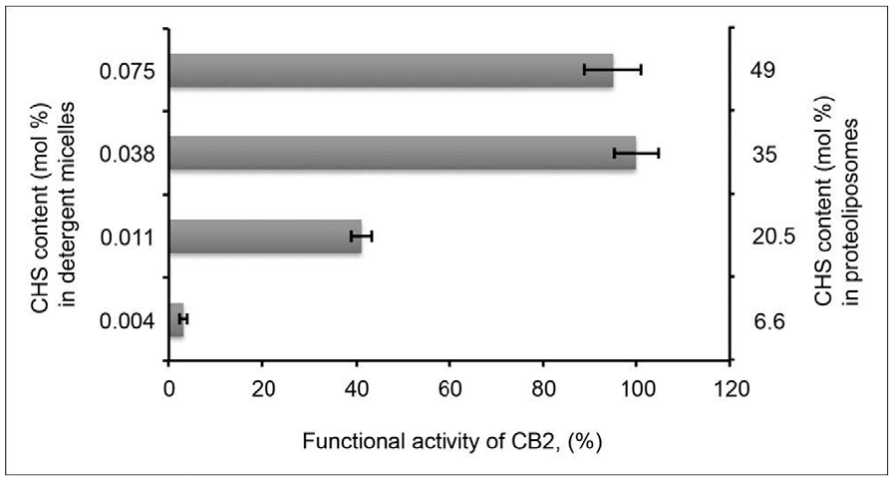
Functional activity of CB_2_ in proteoliposomes. Dependence of functional activity of CB_2_ on CHS content in detergent micelles and in proteoliposomes. The sample with the highest activity within this series (0.1% CHS in micelles) is shown as 100% of activity. The figure depicts data ± SD (error bars) of duplicate measurements from representative experiments (n = 3).

Even a relatively short exposure to detergent micelles without CHS severely destabilizes CB_2_, and only a small fraction of the receptor remained active after 3 hours of incubation at 4°C at these conditions (Fig. 4). The addition of 0.03% (w/v) CHS protected ≥40% of functional receptor, and 0.1% CHS resulted in the maximal level of functional activity although higher concentration of CHS (0.2% w/v) did not aid the recovery of active CB_2_ any further.

However, the proteoliposomes contained different amounts of CHS, ranging from 0 to 50 mol % of total lipid content, depending on the content of CHS in protein preparation used for the reconstitution (Fig 4). Therefore, the observed differences in activation of G proteins could also be attributed to differences in the properties of the lipid matrix of these proteoliposomes, in particular, a different content of CHS that carries a negative charge at physiological pH [19].

#### Discerning stabilizing and activating effects of CHS

The lipids with the negatively charged head group enhance the activation of G proteins by agonist-bound CB_2_, and the content of ∼50–60 mol % of anionic lipids correlates with the maximal levels of activation; note that proteoliposomes contained ∼25 mol% of CHS to assure the functional integrity of the protein [19]. Thus, in order to correctly interpret the results of G protein activation in CHS-containing proteoliposomes, it is necessary to distinguish between the contribution of CHS to stabilization of CB_2_ in micelles and its activating effect on the receptor reconstituted into liposomes.

**Figure 5:**
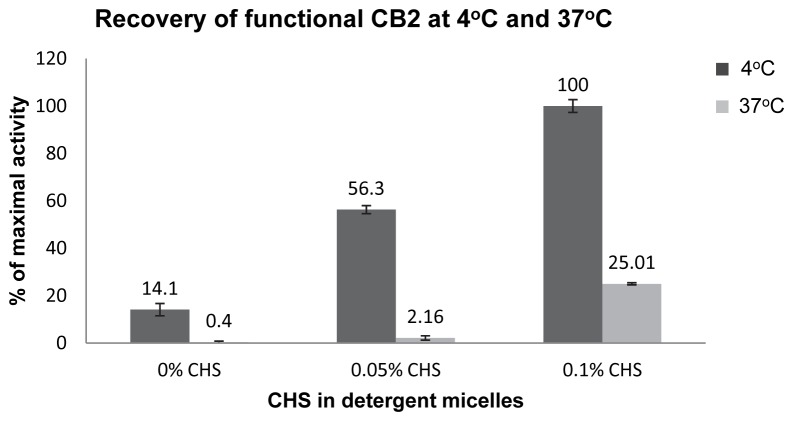
Effect of CHS on stability of CB_2_ in detergent micelles at 4°C and 37°C. Activity of CB_2_ recovered from micelles supplemented with 0.1% CHS at 4°C was set as 100%. Figure presents results of a typical experiment (out of a total of 3), each point is an average of two measurements of the same sample with SD as indicated.

The experimental strategy was essentially the same as outlined in Fig 1,B. The receptor was incubated for 30 min at either 4°C or 37°C in buffers containing different amounts of CHS. Upon incubation, samples were mixed with supplemental lipids to equalize the composition of lipids to POPC:POPS:CHS (50∶25:25 w/w/w). Proteoliposomes were then formed, and the activity of CB_2_ analyzed (Fig. 5). The duration of exposure of protein samples to detergent buffers was 2 hours.

The supplementation of micelles with 0.1% CHS resulted in the highest activity of CB_2_ at 4°C while in buffers without CHS only ∼ 14% of activity was recovered. Incubation at 37°C led to a much more rapid decline of activity: ∼75% of the functional receptor was lost at 0.1% of CHS, and in the absence of CHS the activity was almost entirely lost. Importantly, possible structural perturbations of CB_2_ in detergent micelles without CHS appear to be irreversible since the addition of CHS to CB_2_/DDM/CHAPS micelles just prior to reconstitution of the protein into proteoliposomes did not restore activity.

Thus the TD buffer supplemented with 0.1% CHS can protect a significant fraction of the functional CB_2_ at 4°C for 2–3 days required to complete the chromatographic purification. Typically, the specific activity of CB_2_ purified in the presence of DDM/CHAPS/CHS and reconstituted into proteoliposomes is ∼30–35% of that of the fusion CB_2_-130 in *E. coli* membranes (Fig. S2). The radioligand binding assay performed on the same proteoliposome preparations confirms this and estimates the content of ligand binding-competent receptor at 30–40% [20]. A more prolonged exposure to detergents leads to a gradual decline in activity, and additionally >25% of functional receptor can be lost after one week of incubation in DDM/CHAPS/CHS micelles. These results strongly suggest that an additional stabilization of the receptor in micelles is needed for recovery of fully functional CB_2_.

### Stabilization by Ligands

#### Effects of cannabinoid ligands on stability of CB_2_ in micelles

**Figure 6:**
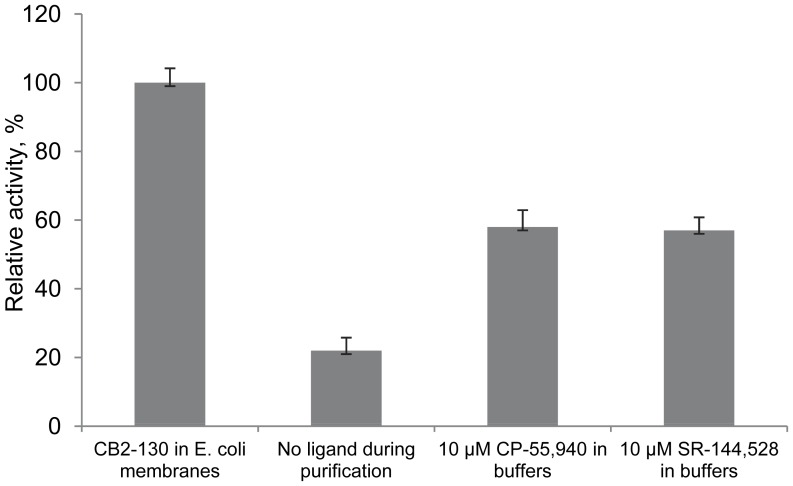
Stabilizing effects of cannabinoid ligands on CB_2_ in detergent micelles. Ligands (CP-55,940 or SR-144,528) at concentration 10 µM were introduced into buffers either through an entire purification procedure or just prior to the reconstitution of the purified CB_2_ into proteoliposomes as indicated. *E. coli* BL21(DE3) membranes expressing fusion CB_2_-130 were used as an activity standard. A quantity of 2 µg of *E. coli* membranes expressing CB_2_-130 or liposomes containing 6 ng of purified, reconstituted CB_2_ were used per reaction. Each point represents an average of duplicate measurements ± SD (error bars) of activity of a representative set of proteoliposome preparation (n = 3).

Since the high affinity ligands have been reported to enhance the stability of several recombinant GPCRs in micelles [10], [39], we studied the effect of cannabinoid ligands on the stability of CB_2_. We first examined the effect of the high affinity agonist CP-55,940 (K_d_∼1.5 nM). The biomass of *E. coli* expressing CB_2_-130 was divided into two equal portions, and the receptor was solubilized and purified in buffers either with or without addition of 10 µM CP-55,940. Purified proteins were then reconstituted into the lipid matrix and the activity analyzed (Fig. 6). The receptor isolated in the presence of CP-55,940 exhibited significantly higher specific rates of activation compared to the protein isolated without ligand, suggesting efficient stabilization by this ligand in micelles.

The stabilizing effect of yet another high affinity cannabinoid ligand, an inverse agonist SR-144,528 was examined in a similar experiment, with some modifications. In this case the fraction of functional receptor could not be accessed directly by measuring the activation rates of G proteins in the presence of an inverse agonist. Moreover, the exchange of the agonist for the inverse agonist in liposomes is inefficient due to the high lipophilicity of both ligands. Therefore, the SR-144,528 used for stabilization of CB_2_ in the course of purification was replaced with CP-55,940 shortly prior to reconstitution of the receptor into proteoliposomes, as described in Materials and Methods.

The activity of CB_2_ purified either in the presence of SR-144,528 or CP-55,940 was equally high, while the protein purified without any ligand was much less active (Fig. 6). These results suggest that both these high affinity ligands are efficient in stabilizing the functional CB_2_ in micelles. At the same time, the low affinity- endogenous agonist 2-arachidonoylglycerol (2-AG) (K_d_ ∼1 µM) was a much weaker stabilizer, and only a small fraction of the receptor isolated in the presence of 10 µM 2-AG remained functional (results not shown).

To test whether the relative content of protein and the high affinity ligand influences stability of CB_2_ in mixed DDM/CHAPS/CHS micelles, the receptor was purified in the presence of an estimated 10-fold excess of CP-55,940 and transferred to a new buffer containing different concentrations of CP-55,940 as described in the Materials and Methods. After an overnight incubation at 4°C, each protein sample was split into two equal aliquots and incubated for additional 30 min at either 4°C or 37°C. The concentrations of ligand were then adjusted in all samples to 30 µM and the activity analyzed upon reconstitution of CB_2_ into the lipid matrix.

**Figure 7:**
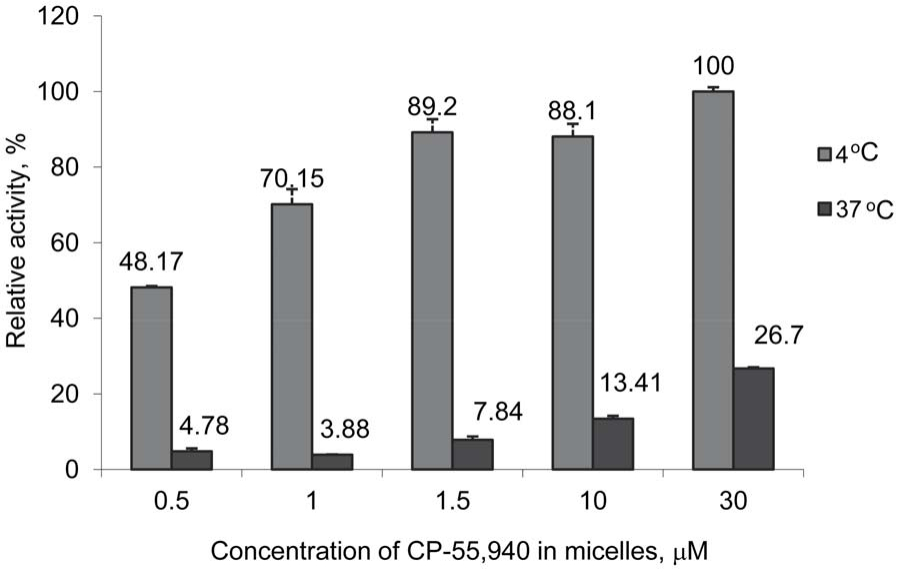
Effect of concentration of CP-55,940 on stability of CB_2_ in micelles. CB_2_-130 dissolved at 1 µM concentration in DDM/CHAPS/CHS (0.1%/0.5%/0.1% w/v) micelles was incubated in the presence of CP-55,940 (at indicated concentrations) at either at 4°C or 37°C, reconstituted into liposomes and its functional activity analyzed as described in the text. The activity of protein recovered after incubation at 4°C with 30 µM CP-55,940 is set to 100%. Each point represents an average of duplicate measurements ± SD (error bars) of a representative proteoliposome preparation (n = 2).

The incubation of 1 µM CB_2_ with CP-55,940 at a protein-to-ligand molar ratio of 0.5 resulted in ∼ 50% loss of functional protein while the concentration of the ligand of 1 µM (molar ratio protein to ligand 1∶1) preserved ≥70% of receptor in a functional form (Fig. 7). An 1.5-fold or higher excess of CP-55,940 was sufficient to fully recover CB_2_ function at 4°C. However, even a short (30 min) exposure to lower (0.5 µM) ligand content at 37°C inactivated about 96% of the receptor in micelles. At this elevated temperature, increasing concentration of the ligand resulted in a progressively higher recovery of functional CB_2_, and a 30-fold molar excess of CP-55,940 protected as much as ∼27% of the receptor.

#### Stabilization of CB_2_ during Expression in *E. coli* Cells

Having established an efficient stabilization of CB_2_ in micelles by CP-55,940 and SR-144,528, we tested whether these high affinity ligands would also aid stability of CB_2_ during its expression in *E. coli* (Fig. S3). The addition of CP-55,940 to the growth medium results in slightly elevated levels of expression (as judged by Western blotting) and a proportional increase in rates of G protein activation. Moderately beneficial effects on expression were also observed with the high affinity agonist, WIN-55,212-2 and inverse agonist SR-144,528, while no effect was detected with the weak agonist 2-AG. These results suggest that high affinity cannabinoid ligands exert a stabilizing effect on CB_2_ during expression.

**Figure 8:**
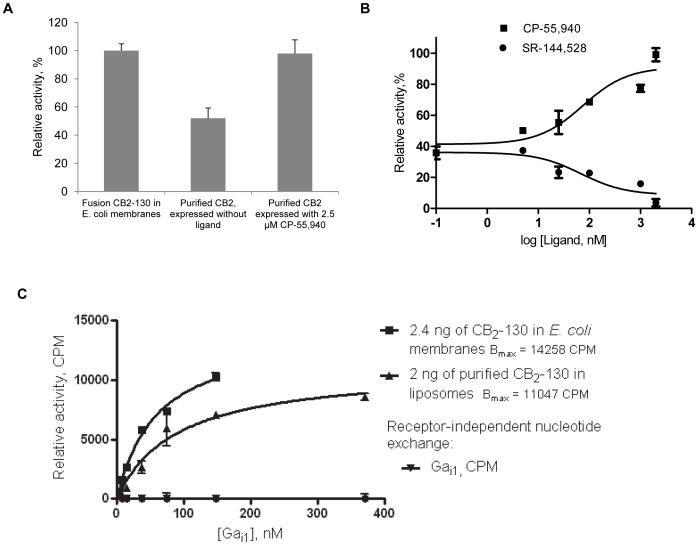
Functional activity of CB_2_ in liposomes. A, Effect of 2.5 µM CP-55,940 in growth media of *E. coli* BL21 (DE3) expressing CB_2_-130 on functional activity of purified and liposome-reconstituted CB_2_. Purified protein was reconstituted into liposomes containing POPC:POPS:CHS (60∶15:25 w/w/w) at a protein-to-lipid ratio 1∶500, and activity measured in the presence of 2 µM CP-55,940 by the G protein activation assay. Membranes of *E. coli* BL21 (DE3) expressing CB_2_-130 fusion protein served as a positive control. Data represents duplicate measurements ± S.D. (error bars) of a representative set of samples (n = 3). 6 ng of the receptor was introduced into the reaction and normalization was performed assuming concentration of CB_2_ of 3 ng per 1 µg of total protein in the *E. coli* membrane preparation. The concentration of CB_2_-130 in membrane preparations was calculated based on a quantitative Western blot probed with anti-CB_2_ antibody, by comparing intensity of the band of fusion MBP-CB_2_ with that of known amounts of purified CB_2_-130 electroblotted onto the same nitrocellulose membrane (not shown). The concentration of CB_2_ in proteoliposome preparations was determined by fluorescence of Alexa-488-labeled CB_2_ added at a ratio of 2∶98 (labeled: unlabeled receptor) to purified CB_2_-130 prior to its reconstitution into liposomes as described in Materials and Methods and in [19]. B, Activation of G proteins by liposome-reconstituted CB_2_ as a function of ligand concentration. Effects of agonist CP-55,940 and inverse agonist SR-144,528. Purified CB_2_-130 stabilized with 2.3-fold molar excess of CP-55,940 was reconstituted into POPC/POPS/CHS (60∶15:25) proteoliposomes. The concentration of CB_2_ in the reaction was 2 nM, and the G protein activation assay performed as described in Materials and Methods. The figure shows duplicate measurements ± S.D. of representative proteoliposome/membrane preparations (n = 3). C, Gα_i1_ saturation of CB_2_-catalyzed GDP/^35^S-γ GTP exchange. ^35^S-γ GTP binding was measured in reactions containing 1.2 nM of CB_2_ in *E. coli* membranes or 1 nM of purified CB_2_-130 reconstituted into proteoliposomes. The contribution of spontaneous nucleotide exchange at a given Gα_i1_ concentration estimated in the absence of CB_2_ was subtracted from total binding. Average of two measurements is presented with S.D. indicated.

An efficient ligand-stabilization of CB_2_ in *E. coli* membranes may, in turn, contribute to higher yield of functional, purified receptor. This was tested by supplementing the growth medium and all buffers for chromatographic purification of CB_2_ with CP-55,940. Indeed, the receptor expressed and purified in the presence of CP-55,940 exhibited higher rates of G protein activation compared to the protein expressed in medium without CP-55,940 (Fig. 8, A), indicating a significant contribution of ligand-stabilization of fusion CB_2_ in *E. coli* membranes to overall recovery of functional purified receptor. Therefore, the maximal yield of purified, functionally active CB_2_ can be achieved through stabilization with the high affinity ligand in bacterial membranes, prior to solubilization of the receptor in detergent micelles. It is practical to synchronize the addition of up to 2.5 µM of stabilizing ligand with the induction of recombinant receptor expression.

#### Functional activity of stabilized CB_2_ reconstituted into lipid bilayers

The lipophilic CP-55,940 is almost quantitatively incorporated into liposomes upon reconstitution of purified CB_2_ from protein-detergent micelles, resulting in ∼2–2.5 molar excess of ligand over the receptor in CB_2_-proteoliposomes as determined by LC-MS (W. Teague et al, unpublished observations). Once in lipid bilayers, hydrophobic ligands cannot be easily removed or replaced which complicates studies of their pharmacological properties on proteoliposome-reconstituted CB_2_.

We examined the functional activity of CB_2_ obtained by expression in *E. coli* in the presence of 2.5 µM CP-55,940, stabilized with 0.1% CHS and 10 µM CP-55,940 in DDM/CHAPS micelles during chromatographic purification and reconstituted into POPC/POPS/CHS proteoliposomes. The presence of an estimated ∼2-fold excess of an agonist over the receptor ensures partial activation of CB_2_ in these liposomes. Treatment with progressively increasing concentrations of CP-55,940 resulted in further >2-fold increase in the rates of activation reaching maximum at ∼100-fold molar excess of ligand (Fig. 8 B). Conversely, treatment with increasing concentrations of the competing inverse agonist SR-144,528 results in a decrease in activation rates demonstrating that the purified receptor can be “cycled” between its inactive (inverse agonist-bound) and active (full agonist-bound) states in lipid bilayers.

We further performed G_αi1_ saturation binding experiments of CB_2_-catalyzed nucleotide exchange (Fig. 8, C). The CB_2_ (1 nM) reconstituted into proteoliposomes was incubated in the presence of CP-55,940 (2 µM) and β_1_γ_2_ subunits of G proteins (500 nM), and titrated with increasing concentrations of purified G_αi1_. The accumulation of the Gα_i1_ bound to the non-hydrolizable ^35^S-γ-GTP progressively increased with the increase in Gα_i1_ reaching saturation at ∼150 nM of the Gα_i1_ subunit. The K_M_ for the Gα_i1_ calculated using a one-site binding equation was ∼90+/−12 nM. A reasonably close value (K_M_ = 61+/−9 nM) was obtained for the fusion CB_2_-130 receptor in *E. coli* membranes (Fig. 8C). Since all components in this assay were provided in a large excess compared to the content of the receptor, the calculated B_max_ value for the Gα_i1_ subunit is proportional to the fraction of functional CB_2_ available for interaction with cognate G proteins. The specific rates of nucleotide exchange were almost identical between these two samples (the calculated B_max_ value for CB_2_ in *E. coli* membranes was∼5940 CPM/ng CB_2_ and for purified CB_2_ in liposomes - 5523 CPM/ng CB_2_, where CPM is proportional to the amount of radiolabel retained by the Gα_i1_ subunit).

In summary, the optimized conditions for stabilization of CB_2_ with high affinity ligands in the course of its expression in *E. coli* and during chromatographic purification in CHS/DDM/CHAPS detergent micelles results in fully functional purified and reconstituted receptor.

### Stabilization by Phospholipids

It has been reported that stability in detergent micelles of several recombinant GPCRs, including β_2_ adrenergic and rat neurotensin receptor can be aided by phospholipids [40], [41]. We, therefore, tested several phospholipids as possible stabilizers for CB_2_ in micelles, with the goal to better control the composition of the lipid matrix of CB_2_-proteoliposomes. The lipids were selected based on their ability to form a mostly fluid phase (in mixtures with POPC), good solubility in detergents, as well as difference in charge of their head group at experimental conditions. POPC, a zwitterionic lipid that is found in plasma membranes at significant concentrations was used as a base lipid, and was supplemented (typically up to 50 mol%) with another lipid whose effects on functional CB_2_ recovery was investigated, namely: POPS, 1-palmitoyl–2-oleoyl-sn-glycero-3-phospho-(1'-rac-glycerol) (POPG), 1,2-dioleoyl-sn-glycero-3-phospho-L-serine (DOPS), 1,2-dimyristoyl-sn-glycero-3-phosphocholine (DMPC), 1-stearoyl-2-oleoyl-*sn*-glycero-3-phosphocholine (SOPC) and cardiolipin (Table S2). Since the replacement of CHS in micelles can be performed within ∼2 hours, for practical reasons the effects of phospholipids were tested by incubating CB_2_ for 2 hours in DDM/CHAPS micelles supplemented with either POPC alone or a mixture of lipids.

**Figure 9:**
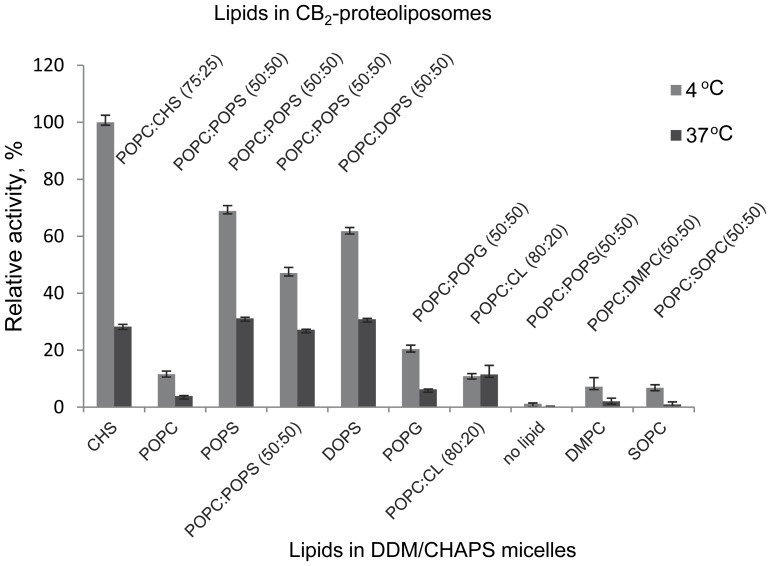
Stabilizing effects of CHS and phospholipids in DDM/CHAPS micelles. Detergent micelles supplemented with lipids as indicated below the graph were incubated either for 2 hours at 4°C (light-shaded bars) or for 1.5 hours at 4°C followed by 30 min at 37°C (dark-shaded bars). The composition of micelles was adjusted as indicated (above the graph) and the protein reconstituted into proteoliposomes on mini-spin detergent absorbent columns (Pierce). The functional activity was measured by the G protein activation assay as described in Materials and Methods. Purified CB_2_ reconstituted from CHS/POPC/DDM/CHAPS micelles (final composition of liposomes: CHS/POPC 25∶75 mol/mol) was used as an activity standard. Presented are average values of duplicate measurements ± S.D. (error bars) from representative experiments (n = 3).

The replacement of lipids, temperature treatment, addition of supplementing lipids, and reconstitution were performed essentially as outlined in Fig. 1,B and described in Materials and Methods. It should be noted that G protein activation rates depend not only on the functional integrity of GPCR but also on lipid composition [19], [42], [43]. Therefore, the differences in G protein activation rates presented in Fig. 9, A (light-shaded bars) do not necessarily report survival rates of CB_2_ in the micellar phase in the presence of different lipids but rather a combined effect of these lipids on protein survival, reconstitution and G protein activation. However, we can directly compare the effect of lipids on protein survival by adjusting the lipid composition just prior to reconstitution.

A second set of samples was tested by raising temperature in the micellar state to 37°C for 30 min before reconstitution followed by measurements of G protein activation (Fig. 9, dark-shaded bars) to probe for a decline of activation rates under a controlled challenge. Receptor stabilized with CHS in micelles (content of lipids POPC/CHS: 75∶25 mol% in liposomes) was used as an activity standard in both sets of samples.

The effects of lipids with 16∶0–18∶1 acyl chain, namely: POPC, POPS and POPG, were first studied. The functional activity of CB_2_ recovered after a 2-hour incubation at 4°C in DDM/CHAPS micelles supplemented with 0.1% POPC was about one order of magnitude lower than that of the receptor incubated in the presence of CHS. Considering that ∼50% of negatively charged lipids in proteoliposomes are likely to produce the highest rates of G protein activation by CB_2_ [19], the protein incubated in the presence of POPC was supplemented with POPS just prior to the reconstitution, such that the lipid content of these proteoliposomes became: POPC:POPS (50∶50). However, on average the rates of G protein activation by receptor isolated from CHAPS/DDM/POPC micelles were less than 10–15% of that of the receptor stabilized by CHS suggesting poor recovery of functional protein from POPC-containing micelles. These results were further supported by ^2^H-CP-55,940 NMR ligand binding studies that indicated very poor ligand binding on a POPC-stabilized receptor. CHS also performed much better at 37°C: as much as 28.3% of activity was recovered from the CHS/DDM/CHAPS micelles compared to 3.9% from POPC/DDM/CHAPS micelles.

A comparison of POPC and POPS indicates that presence of POPS in the micellar phase yields reconstituted CB2 capable of activating G proteins at much higher rates both at 4°C and 37°C. A mixture of POPC/POPS (50∶50 w/w) was almost as efficient as the POPS alone. The estimates, by G protein activation rates, of the recovery of functional protein from POPC:POPS micelles were further supported by the ^2^H-CP-55,940 NMR ligand binding studies that demonstrate a very high recovery of ligand binding-competent receptor at these conditions. Interestingly, while the functional activity of CB_2_ incubated in the presence of CHS was slightly higher than that of POPS at 4°C, there was no significant difference between these two lipids at 37°C. At the same time, the receptor incubated in micelles without any lipid was almost completely inactive.

Since these results suggest that the presence of negatively charged headgroup may contribute to protection of CB_2_ in micelles, the effects of two other anionic phospholipids POPG and POPS were compared. POPG turned out to be significantly less potent than POPS at 4°C in activating G protein, and an even more significant difference between these two lipids was observed at 37°C. While POPS or mixtures of POPS/POPC (50∶50) were as effective as CHS at 37°C, G protein activation rates from reconstituted CB_2_ in POPG-micelles were approximately 4 times lower.

Cardiolipin (CL) of *E. coli*, another anionic lipid (that carries two negative charges per molecule), when provided in a binary mixture with POPC (80∶20 w/w) at 4°C did not improve G protein activation by CB_2_, compared to POPC. However, those low rates did not decline any further with an increase of incubation temperature to 37°C.

Effects of variations in both *sn*-1 and *sn*-2 acyl chains on stability of CB_2_ were examined by testing zwitterionic lipids with choline headgroup: POPC, DMPC and SOPC, whose phase transition temperatures cover a range from −2 to +23°C (Table S2). However, DMPC and SOPC, when introduced into micelles as binary mixtures with POPC (50∶50 w/w) yielded very low G protein activation rates on CB_2_ (Fig 9, A) and, therefore, were not selected for further work.

The effect of the *sn*-1 acyl chain of anionic lipids was studied by comparing stabilizing effects of POPS (16∶0–18∶1) and DOPS (18∶1). DOPS yielded only slightly lower G protein activation rates than POPS at 4°C, and was almost equally effective at 37°C (Fig. S4). Since the composition of proteoliposomes used for measurements of the functional recovery of CB_2_ in this experiment was identical for all samples (POPC:POPS:DOPS, 50∶25:25), the measured G protein activation rates are likely to represent the relative stabilizing effects of POPS and DOPS in micelles rather than their combined effects on stabilization of CB_2_ in micelles, reconstitution and activation of G proteins. They also suggest the importance of the anionic headgroup for stabilization of CB_2_ in micelles.

**Figure 10:**
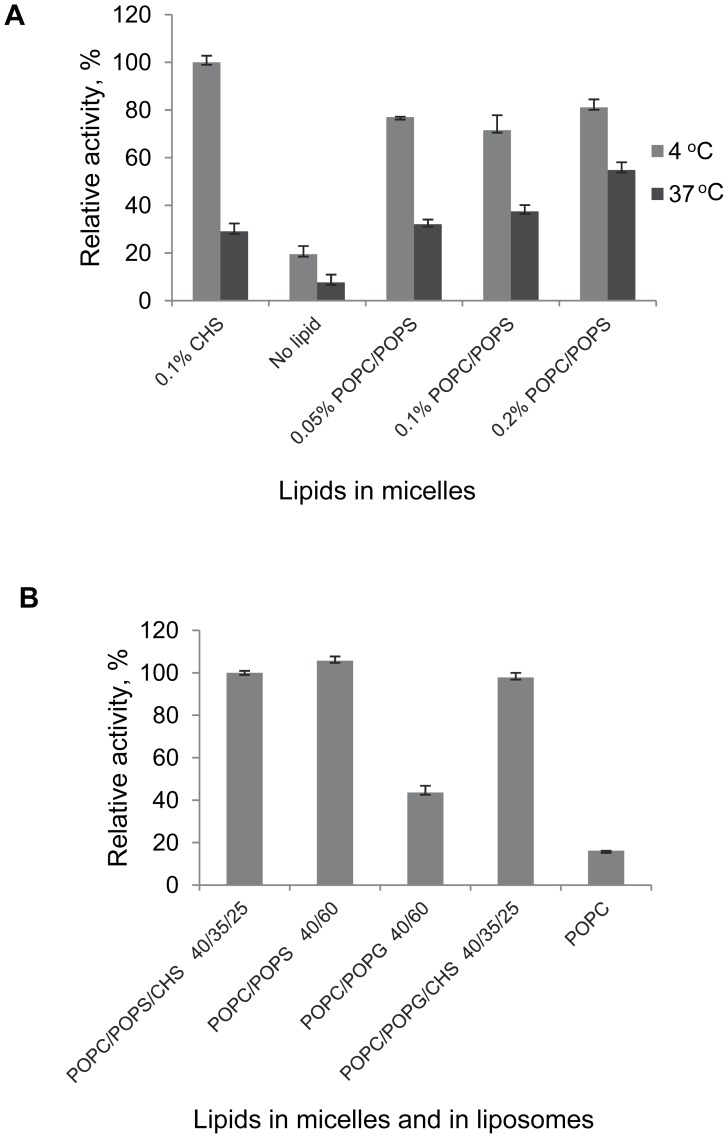
Stabilizing effects of POPC:POPS (1∶1, w/w) in micelles. A, Lipids were added to DDM/CHAPS (0.1%/0.5%) micelles at concentrations indicated. Upon incubation samples were supplemented with POPC:POPS dissolved in 1% CHAPS so that the final protein-to-lipid ratio was the same in all samples (1∶500 mol/mol). Upon reconstitution on a mini-spin detergent-absorbent columns the activity of CB_2_ was determined by the G protein activation assay and reported as % of the maximal activity measured for this series of samples. The liposomes-reconstituted receptor (0.1% CHS in micelles, POPC/POPS/CHS 60∶15:25 in liposomes) exhibiting the highest levels of activation in this experiment was used as an activity standard. B, Purified CB_2_ in DDM/CHAPS/CHS micelles was captured on Ni-NTA, detergent buffer rapidly exchanged to DDM/CHAPS containing 0.4% of lipids of indicated composition, protein eluted with imidazole and liposome-reconstituted by rapid dilution. Functional activity of CB_2_ reconstituted into POPC/POPS/CHS matrix is set as 100% of activity. Figures depict data ± SD (error bars) of duplicate determinations from representative experiments (n = 2-3).

Clearly, not only the type but also the concentration of phospholipids in micelles may contribute to the efficiency of stabilization. This was tested by analyzing the recovery of functional CB_2_ from DDM/CHAPS micelles supplemented with variable amounts of POPC:POPS (50∶50, w/w). At the concentration of 0.05% these lipids exhibited significant protective effect (Fig. 10, A) while increase in lipid concentrations up to 0.2% w/v did not significantly improve G protein activation at these conditions (2 hours incubation, 4°C). However, at 37°C an increase in the lipid content of micelles correlated with an increase in the recovery of functional protein.

We then tested whether a higher total lipid concentration in micelles combined with a shorter incubation time of CB_2_ in micelles will further aid the recovery of functional receptor. The purified receptor was incubated in DDM/CHAPS (0.1%/0.5%) micelles containing 0.4% (w/v) of supplementing lipids as indicated in Fig. 10, B, and reconstituted into liposomes within 1 hour of the start of the buffer exchange. In this experiment the reconstitution of CB_2_ into proteoliposomes was performed by the rapid dilution method, and the lipid matrix (with the exception of POPC-sample) contained 60% of anionic lipids, allowing normalization for the likely effect of negatively charged lipids on G protein activation [19]. The receptor recovered from micelles supplemented with POPC/POPS (40∶60) was as active as CB_2_ from POPC/POPS/CHS (40∶35:25) micelles, pointing to a significant stabilizing effect of the phospholipid with the serine headgroup. POPG, on the other hand, was significantly less effective than either CHS or POPS, and rates of G protein activation were only 40% of that of POPC/POPS-containing sample.

In summary, the negatively charged lipids in DDM/CHAPS micelles exert a stabilizing effect on CB_2_, with CHS being the most efficient followed by POPS, DOPS, and significantly lower effects from POPG, and cardiolipin (in this order). Among anionic phospholipids tested, stabilization with lipids containing the serine headgroup appears to be the most efficient. Uncharged phospholipids: POPC, SOPC, DMPC and 1,2-dioleoyl-sn-glycero-3-phosphoethanolamine (DOPE), were least effective.

### Ligand Binding to CB_2_ by Solid-state NMR

The G protein activation assay provides a relative measure of the functional activity of the receptor. The ligand binding, on the other hand, is a more direct way to quantify the content of functional CB_2_. Since these two functional tests assess somewhat different features of the receptor, it is advantageous to ascertain whether the estimates of the content of functional CB_2_ provided by these methods agree. Since the CB_2_ has to be stabilized with the high affinity ligand, it is technically challenging to perform the radioligand binding assay either in micelles or on purified CB_2_ reconstituted into liposomes. Therefore, the ligand binding was performed using an ^2^H-labeled agonist, CP-55,940-*d*_6_, in ^2^H MAS NMR experiments as described elsewhere in detail [19]. The assay requires significant quantities of ^2^H-labeled ligand and a large amount of purified receptor and, therefore, was performed only on a few select samples.

^1^H-CP-55,940 used for stabilization of CB_2_ during expression and purification was displaced by the CP-55,940-*d*_6_ as described in Materials and Methods, and the labeled ligand was partially competed off the binding pocket of the receptor by the 10-fold molar excess of unlabeled CP-55,940. Upon introduction of the unlabeled ligand, the fraction of the unbound CP-55,940-*d*_6_ increased, and the intensity of ^2^H signal increased accordingly (Fig. S5, A). When purified in the presence of 0.1% CHS and 10 µM CP-55,940≥90% of the receptor maintained the ligand binding ability. In contrast, when CB_2_ was reconstituted from micelles in which CHS was replaced with POPC, the ^2^H signal of CP-55,940-*d_6_* showed virtually no change upon introduction of the excess of unlabeled CP-55,940 (Fig. S5, B) confirming poor stabilizing effect of POPC, as observed by the G protein activation studies.

The competition ligand-binding on CB_2_ stabilized in the presence of POPC:POPS (40∶60, w/w) demonstrated that as much as ∼82% of the receptor retained ligand binding competence upon reconstitution into proteoliposomes (Fig. S6). This result correlates well with estimates, by the G protein activation assay, of the fraction of functional CB_2_ stabilized with a mixture of POPC and POPS 40∶60 (Fig. 10, B). Therefore, POPS appears to be only slightly less efficient for stabilization of CB_2_ compared to CHS, while POPC is a much weaker stabilizer.

### Stability of CB_2_ in Lipid Bilayers

Lipid bilayers stabilize GPCRs better than the detergent micelles [7], and a reconstitution into liposomes is an efficient way of protecting the purified, functional receptor. To examine stability of CB_2_ in lipid bilayers, the CB_2_ in proteoliposomes (POPC:POPS:CHS, 60∶15:25, w/w/w) matrix were stored at +4°C and –80°C for several weeks, samples withdrawn periodically, and activity analyzed. The receptor is very stable at -80°C displaying constant activity over a period of several weeks (Fig. S7). When incubated at 4°C, CB_2_ lost ∼7.5% of activity after 1 week, and another 10–12% - after two weeks of storage. However, there could have been an underestimation of receptor activity because multilamellarity of proteoliposomes may have increased during the prolonged storage.

#### Temperature stability

The stability of CB_2_ in lipid bilayers was assessed at increasing temperatures. While the thermal denaturation of proteins can be studied with biophysical techniques that measure changes in secondary and tertiary structure upon temperature treatment, it is not known at present which structural features of CB_2_ can be regarded as truly representative of its “functional fold”. In this respect, measurement of receptor function is much more relevant for characterizing stability.

**Figure 11:**
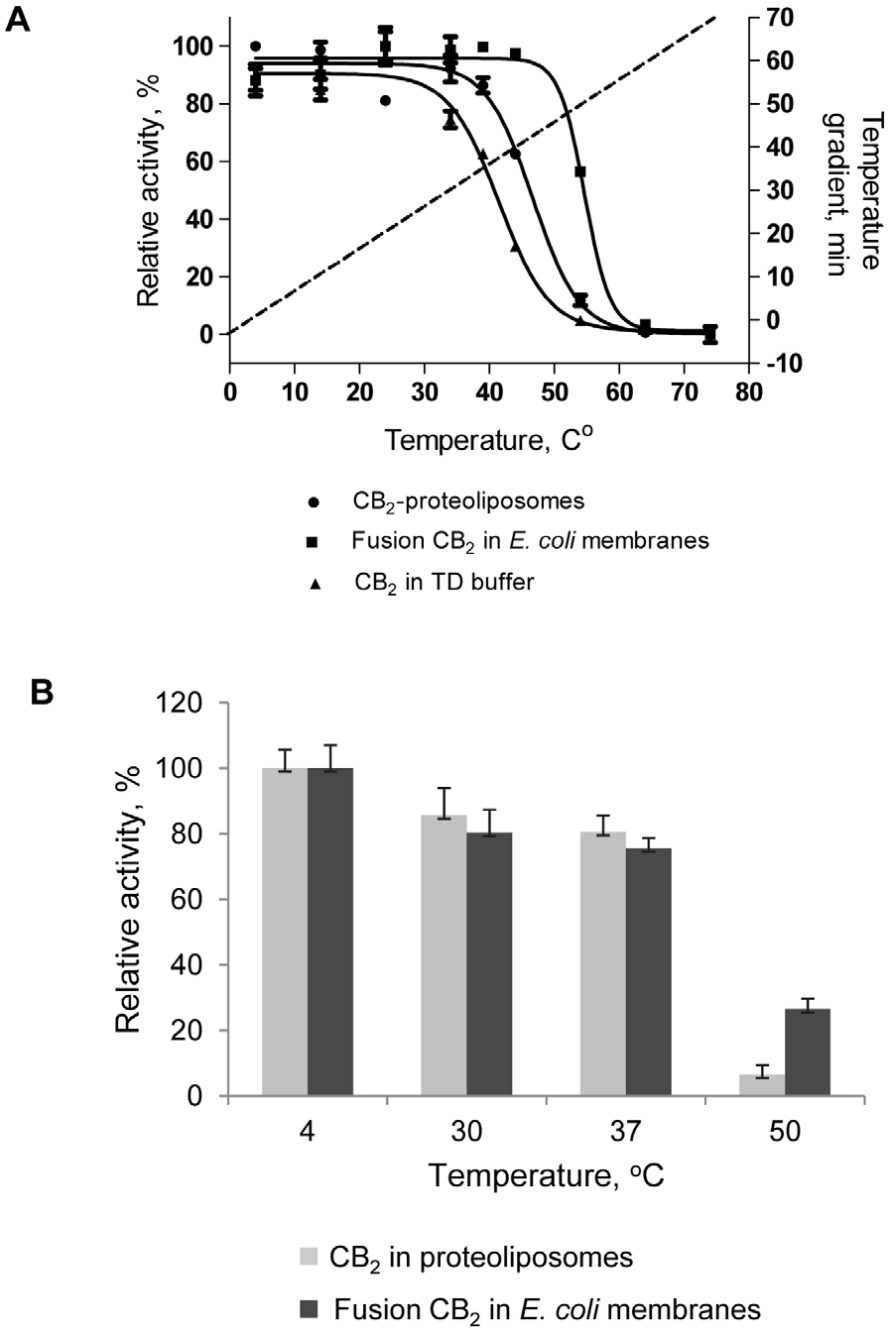
Stability of CB_2_ in lipid bilayers. A, Temperature-induced unfolding of CB_2_ in detergent micelles and lipid bilayers. For stability studies in micelles the purified CB_2_-130 in TD buffer supplemented with 10 µM CP-55,940 was subjected to a temperature gradient from 4°C to 74°C at a rate of 1°C/min, 10 µg protein samples withdrawn at indicated time points, mixed with 100 µg lipids POPC/POPS (4∶1 w/w) in 1% CHAPS and diluted 110-fold into cold 10 mM MOPS buffer under vigorous stirring. The activity of CB_2_ was analyzed by measuring the G protein activation rates as described in Materials and Methods. For measurement of thermostability in lipid bilayers either CB_2_-proteoliposomes or membrane preparations harboring fusion CB_2_-130 were suspended in 10 mM MOPS buffer at a concentration of CB_2_ 0.5 ng/µL, subjected to treatment with linear temperature gradient, and analyzed by G protein activation assay. Dotted line depicts the temperature gradient profile. Figure depicts data ± S.D (error bars) of duplicate measurements from representative experiments (n = 3). B, Temperature stability of CB_2_ in proteoliposomes and *E. coli* membranes. Either purified CB_2_ receptor reconstituted into POPC:POPS:CHS bilayers or fusion CB_2_-130 in *E. coli* membranes was incubated for 30 min at the temperatures indicated, and the G protein activation assay performed. 4 ng of CB_2_ was used in every reaction and measurements were performed upon addition of 2 µM of CP-55,940 to all samples. Data ± S.D. (error bars) of duplicate measurements from representative experiments (n = 3) are presented.

The experiment was performed in two different formats. First, the purified CB_2_ reconstituted into proteoliposomes consisting of POPC/POPS/CHS (60∶15:25) was heated from 4°C to 74°C at a rate of 1°C/min, samples withdrawn at indicated time points and analyzed by the G protein activation assay (Fig. 11, A). Under these conditions, 50% of CB_2_ loses its ability to activate G proteins at a temperature (T_50_) of 46.7+/−1.9°C. For comparison, the purified CB_2_ in TD micelles (0.1%DDM, 0.5% CHAPS, 0.1% CHS) supplemented with 10 µM CP-55,940 was subjected to the same temperature protocol. Samples drawn at indicated intervals were reconstituted into POPC/POPS/CHS liposomes, and their activity analyzed. As expected, the thermal stability of CB_2_ in micelles was lower, with a loss of 50% of activity at a T_50_ of 41.5+/−1.5°C. The fusion CB_2_-130 in *E. coli* BL21(DE3) membranes subjected to the same temperature treatment is more stable compared to the purified receptor, with a T_50_ of 54.7+/−2.0°C. These results might suggest a stabilizing effect on CB_2_ from the MBP fusion partner. However, it needs to be pointed out that the experiments on purified CB_2_ in proteoliposomes were conducted in POPC/POPS/CHS lipid matrix while experiments on fusion CB_2_-130 were conducted in *E. coli* membranes. The differences in lipid composition, protein concentration between artificial bilayers and *E. coli* membranes as well as the presence of other proteins in *E. coli* membranes could have played a role.

For practical applications it is important to know the stability of the receptor exposed to different temperatures for a fixed period of time. This was done by keeping CB_2_-containing proteoliposomes at various temperatures for a period of 30 min with quantification of CB_2_ activity by measurement of G protein activation (Fig 11, B). While the purified and reconstituted receptor has long-term stability at 4°C, incubation at 30 or 37°C results already in about 10% loss. Higher temperatures were more detrimental; after 30 min treatment at 50°C less than 10% of receptor remained active.

The fusion CB_2_ protein in *E. coli* membranes exhibited a similar response to treatment at 30°C or 37°C with only a slight decline of activity. But in difference to CB_2_, the fusion-CB_2_ appears to be more stable at 50°C with about 30% of the protein remaining functional after 30 min (Fig. 11, B).

To summarize, the reconstitution of the purified, detergent-solubilized CB_2_ into lipid bilayers (liposomes) significantly improves stability of the receptor. Active fold of CB_2_ in proteoliposomes at +4°C (or lower temperatures) can be maintained for prolonged periods of time, necessary to study the structure and function of this protein in artificial bilayers by a range of biophysical methods.

## Discussion

By screening a large number of nonionic and zwitterionic detergents, we optimized conditions for solubilization of CB_2_ from *E. coli,* and determined that a combination of DDM and CHAPS was the most efficient in solubilizing as well as adequate in preserving the functional fold of the receptor. Furthermore, for the purified CB_2_, we optimized a reconstitution procedure using either CHAPS or LDAO as dominant detergents, and demonstrated that the fully functional receptor was incorporated with high yield into lipid bilayers.

Our efforts to prepare the fully functional recombinant CB_2_ focused on the stabilization of receptor in detergent micelles. Summary of stabilization conditions are presented in Tables 1, 2, 3 and 4. The CHS was found to be the most efficient stabilizer, although this compound is not required for functioning of CB_2_ in membranes. In fact, CB_2_ is fully functional in lipid bilayers without CHS although the activation rates may be significantly enhanced by addition of certain negatively charged lipids [19]. While we previously reported the addition of CHS to solubilization and purification buffers for CB_2_ [5], [20], no systematic study of its stabilizing effect was performed at the time. In particular, it was not clear whether this cholesterol-like compound could be excluded (even for a short period of time) from the detergent buffers without compromising the structural integrity of the receptor. This is of importance since an ability to control the composition of lipid matrix is critical for studies of functioning of CB_2_ in lipid bilayers.

**Table 1:** Stabilization of CB_2_ (summary). Expression and purification of functional CB_2_.

Purification step	Ligands in growth medium	Ligands in purification buffers	CHS in DDM/CHAPS micelles	Yield of purified CB_2_ (estimated)	% of functional receptor	Reference
Membrane preparation	none	none	n/a	>400 µg/L	100% (G protein activation, ligand binding	[20], this study
Purified CB_2_	none	none	No CHS	n.d.	0	Fig. 8A & S3
“-“	none	none	0.1% CHS	∼250 µg/L	25–35% (by ligand binding)	[20]
“-“	none	10 µM CP-55,940	0.1% CHS	∼250 µg/L	>60% (G protein activation)	Fig. 6
“-“	2.5 µM CP-55,940	10 µM CP-55,940	0.1% CHS	∼250 µg/L	≥90% (G protein activation)	Fig.8, S5,
“-“	none	10 µM SR-144,528	0.1% CHS	∼250 µg/L	>60% G protein activation)	Fig. 6

**Table 2:** Reconstitution of purified CB_2_ into proteoliposomes.

Dominant detergent	Lipid matrix	Relative activity (by G protein activation)	Reference
1% CHAPS	POPC/POPS/CHS	95%	Fig. 2
1% LDAO	“-“	100%	“-“
1% OG	“-“	71%	“-“

**Table 3:** Stabilization of CB_2_ in DDM/CHAPS micelles.

	Concentration		Activity		Reference
	CHS	Ligand	%, by G protein activation	%, by CP-55,940-d_6_ binding	
Effect of CHS					
	0	10 µM	0		Fig. 4, S2
	0.05%	10 µM	39		Fig. 4, S2
	0.1%	10 µM	100	≥90	Fig. 4, S2, S5
Effect of ligand CP-55,940					
CB2:ligand 2∶1	0.1%	0.5 µM	48		Fig. 7
CB2:ligand 1∶1.5 to 1∶30	0.1%	30 µM	90–100		Fig. 7
Effect of phospholipids					
POPC/POPS (40∶60)	0.4% (w/v)		100	82	Fig. 10
POPS	0.2% (w/v)		75–80		Fig. 10
POPC	0.2% (w/v)		10–12	≤5	Fig. 9, 10, S5

**Table 4:** Stability of CB_2_ in liposomes and in *E. coli* membranes.

	Conditions of treatment	Residual activity	Reference
Stability in liposomes	4°C or -80°C, 2 weeks	Up to 100%	S7
	37°C, 30 min	∼80%	Fig. 11
	50°C, 30 min	∼7%	Fig. 11
Stability in *E. coli* membranes	-80°C, several years	Up to 100%	This study
	37°C, 30 min	∼76%	Fig. 11
	50°C, 30 min	26%	Fig. 11

Here we demonstrate that the 0.1% (w/v) concentration of CHS is optimal for stabilization of the functional fold of CB_2_ in detergent micelles at 4°C. Exclusion of CHS from micelles results in a rapid and irreversible loss of function of CB_2_.Interestingly, a recently published study on the recombinant human adenosine A_2_a receptor suggests that almost the same content of CHS (0.11%, w/v) in DDM/CHAPS/CHS micelles was optimal for keeping this protein in a functional, ligand-binding competent form [44]. It was proposed that the size and the shape of CHS-containing micelles plays a major role in stabilization of this GPCR.

However, the presence of CHS in detergent micelles is beneficial but not sufficient to protect the receptor and, on average, no more than 30–40% of CB_2_ retained functionality after several days of incubation in micelles with CHS. Therefore, we explored additional approaches to stabilization and determined that the high affinity cannabinoid ligands CP-55,940 and SR-144,528 significantly improve the yield of functional CB_2_, both during its expression in *E. coli* BL21 cells and upon solubilization in detergent micelles. Taking advantage of a concerted action of CHS and a high affinity ligand the CB_2_ receptor stabilized in either ground (SR-144,528) or activated (CP-55,940) functional conformations can be prepared. Stabilization by the high affinity ligands was previously reported for other GPCRs including β2 adrenergic receptor [45], CXCR4/δ opioid receptor [46] and others [47].

A slightly higher than equimolar ratio between the CP-55,940 and receptor was required to preserve the functional CB_2_ in micelles at 4°C. On the other hand, the increased concentration of ligand clearly correlated with the improved stability of CB_2_ at 37°C, likely due to the change in activation energy and correspondingly higher rates of exchange between the ligand molecules at the binding pocket of CB_2_ and (free) micelle-solubilized CP-55,940.

Studies of the lipid-CB_2_ interaction require a precise control over the composition of lipid matrix. This can be achieved by replacing the CHS with yet another stabilizing lipid prior to reconstitution. In this respect, the derivatives of phosphatidylserine were the most efficient for stabilization while other negatively charged at physiological pH lipids (POPG or cardiolipin) did not perform that well. The uncharged lipids, such as POPC, SOPC, DMPC or DOPE were even less effective. These results suggest that the presence of the negatively charged head group may contribute to the stabilization, and that the phospatidylserine moiety seem to be the most effective in this respect. It is intriguing that the negatively charged lipids also exert substantial beneficial effect on activation of CB_2_ in lipid bilayers [19]. For the limited number of lipids tested there does not seem to be a clear correlation between the structure of acyl chains or the phase transition temperature of a phospholipid and its stabilizing effect in micelles.

The stabilization by synthetic phospholipids has been reported earlier for the β_2_ adrenergic receptor and the rat neurotensin receptor [40], [41]. Our results demonstrate that not only the type of the phospholipid but also its concentration in micelles affects the stability of CB_2_. At 4°C the 0.05% (w/v) of lipid was already sufficient to achieve a significant effect, and higher concentrations of phospholipids POPC/POPS (0.4% w/v) were required to recover a fully functional receptor. At a higher temperature (37°C) the stabilizing effect increased almost proportionally with the concentration of lipid.

As expected, the receptor is much more stable upon reconstitution into lipid bilayers, losing only a small fraction of activity upon storage for several weeks at 4°C. These results are of importance since many biophysical studies requiring long acquisition times can be performed on a functional receptor in liposome. We demonstrate higher temperature stability of fusion CB_2_ in *E. coli* membranes compared to that of the purified CB_2_ in artificial lipid bilayers which may be indicative of certain stabilizing properties of MBP fusion partner and/or differential effects of lipid/protein composition of the membrane. Experiments are underway to assess the stability of CB_2_ in bilayers of various lipid compositions.

In summary, we report insights into efficient stabilization of the cannabinoid receptor CB_2_ in detergent micelles and in lipid bilayers that may prove instrumental for studies of structure-function relationship of this pharmacologically important GPCR. We believe that our experimental strategy for assessing functional structure and stability that relies on measuring G protein activation by the purified receptor reconstituted into liposomes may be applicable to other recombinant GPCRs, especially to those that interact with highly hydrophobic ligands and thus are not readily amenable for conventional ligand binding assays.

## Materials and Methods

### Chemicals and Reagents

CP-55,940 was purchased from Tocris Cookson Inc. (Ellisville, MO). Labeled CP-55,940-*d_6_* was synthesized and generously provided by Drs. Kejun Cheng and Kenner C. Rice (NIH). The CB_2_ antagonist SR-144,528 was obtained from the National Institute on Drug Abuse (Research Triangle, NC). The HisProbe-HRP kit for detection of polyhistidine fusion proteins was obtained from Thermo Scientific (Rockford, IL), the Ni–NTA resin from Qiagen (Valencia, CA), and the StrepTactin Macroprep resin from EMD-Novagen (Gibbstown, NJ). The antibodies against CB_2_ were purchased from Cayman Chemicals (Ann Arbor, MI). Cholesteryl hemisuccinate Tris salt (CHS), the detergents 3[(cholamidopropyl) dimethylammonio]-1-propanesulfonate (CHAPS) and *n*-dodecyl-β-D-maltoside (DDM) were obtained from Anatrace (Maumee, OH). *N*-Octyl-β-D-glucopyranoside (OG) was purchased from EMD-Calbiochem (San Diego, CA). *N,N*-Dimethyldodecylamine N-oxide (LDAO) was purchased from Sigma (St. Louis, MO). The lipids 1-palmitoyl-2-oleoyl-*sn*-glycero-3-phosphocholine (POPC) and 1-palmitoyl-2-oleoyl-*sn*-glycero-3-phosphoserine (POPS) were obtained from Avanti Polar Lipids, Inc. (Alabaster, AL). ExtractiGel D Detergent Removing Gel was purchased from Thermo Scientific (Rockford, IL). SM2 Biobeads were procured from EMD-Calbiochem (San Diego, CA). DilC18 and Alexa Fluor 532 were purchased from Invitrogen, Inc. (Carlsbad, CA). β-methyl cyclodextrin was purchased from Sigma (St. Louis MO).

Membranes expressing CB_2_ protein in CHO cells were from Perkin Elmer, Waltham, MA.

### Expression and Purification of the Recombinant CB_2_

Expression and purification of CB_2_ was previously described [5], [20]. CB_2_-130 was expressed as a fusion with maltose binding protein followed by the TEV protease recognition site at the N-terminus, and a decahistidine tag at the C-terminus, using an expression construct pAY-130. Expression was performed in *E. coli* strain BL21 (DE3) (EMD Millipore, Billerica, MA). Recombinant CB_2_ was solubilized in a buffer containing 50 mM Tris pH 7.5, 200 mM NaCl, 30% (v/v) glycerol, and supplemented with 1% (w/v) DDM, 0.5% (w/v) CHAPS and 0.1% (w/v) CHS. Fusion protein was immobilized on a Ni-NTA resin, washed with buffer A (50 mM Tris pH 7.5, 200 mM NaCl, 30% (v/v) glycerol, 0.1% (w/v) DDM, 0.5% (w/v) CHAPS and 0.1% (w/v) CHS) and eluted with buffer B (buffer A supplemented 250 mM imidazole). The fractions containing fusion protein were pooled, dialyzed for 4 hours against buffer C (50 mM Tris pH 7.5, 100 mM NaCl, 15% (v/v) glycerol, 0.1% (w/v) DDM, 0.5% (w/v) CHAPS, 0.1% (w/v) CHS), and the protein was treated with TEV protease (1 mg of protease per10 mg of CB_2_ fusion, 4°C, 4 hours) to remove the fusion partners. The resulting CB_2_ was further purified on a hand-packed StrepTactin Macroprep column. After elution with 5 mM desthiobiotin the fractions containing purified CB_2_ were pooled and concentrated in centrifugal spin concentrators (Orbital Biosciences) to a final protein concentration of 1–2 mg/mL. The concentrated protein solution was divided into aliquots 100 µL, frozen in liquid nitrogen, and stored at −80°C until use.

### Preparation of Membranes

Membranes from the *E. coli* cells expressing recombinant CB_2_ were prepared according to the previously published protocol [5] and stored at −80°C until use.

### Exchange of Detergents, Lipids and Ligands

Typical protocol for exchange of detergents solubilizing CB_2_ was as follows. 200 µL of Ni-NTA resin (GE Healthcare) equilibrated in buffer A (50 mM Tris-HCl, pH 7.5 supplemented with 30% glycerol, 200 mM NaCl and detergents 0.5% CHAPS, 0.1% CHS and 0.1% DDM) and re-suspended in the same buffer were dispensed into the 1.5 mL Eppendorf tubes equipped with the centrifuge tube filters Spin-X (Costar) and centrifuged at 1200 rpm for 1 min to remove the excess buffer. 200 µL of CB_2_-130 solution (containing 1% of AlexaFluor 488- labeled CB_2_) was added to the resin, and columns centrifuged at 1200 rpm for 1 min. The flow-through was collected and re-applied onto the resin two more times, to ensure efficient binding of the His-tagged protein. 200 µL of solution of the replacing detergent buffer in Tris-HCl pH 7.5 supplemented with 30% glycerol and 200 mM NaCl was applied to the resin, and the column centrifuged at 1200 rpm for 1 min. The column was washed with the solution of detergent 5 more times to ensure efficient detergent exchange. Finally, protein was eluted with 4x 200 ml of detergent buffer supplemented with 250 mM imidazole. The elution fractions were combined, and protein concentrated in Ultrafree centrifugal filter tubes (Millipore). The recovery of the protein was determined by measuring fluorescence of the resulting fraction.

Exchange of lipids in detergent micelles was performed as follows. 200 µL of Ni-NTA resin was packed into the 5 mL disposable column, and equilibrated with buffer A supplemented with 10 µM CP-55,940. 200 µg of purified CB_2_-130 and 2 µg of AlexaFluor 488-labeled CB_2_-130 were diluted to 400 µL with buffer A containing 10 µM CP-55,940 and passed three times through the column. Then 800 mL x5 of the exchange buffer containing 50 mM Tris pH 7.5, 200 mM NaCl, 10% glycerol, 1% CHAPS, 10-fold molar excess over the protein of CP-55,940 and 4 mg/mL of the lipid mixture were passed through the column. CB_2_ was eluted from the resin by applying 500 µL of the exchange buffer supplemented with 250 mM imidazole. The yield of the protein and lipids was determined by fluorescence measurements of the trace amounts of the fluorescently labeled CB_2_ and lipids. Reconstitution was performed by rapidly diluting the eluted protein-lipid-detergent mixture 100-fold into cold PBS.

The exchange of ligands was performed essentially the same way as the exchange of lipids. The protein isolated in the presence of SR-144,528 was immobilized on a Ni-NTA resin and buffer was exchanged to a new one, supplemented with 10 µM of CP-55,940. Elution of CB_2_ and reconstitution were performed as described above.

### Reconstitution of CB_2_ into Liposomes

Eighty mg of POPC and 20 mg of POPS (2 mL of 10% solution) were added to a siliconized test tube so that the final weight ratio of POPC and POPS was 4∶1. To this mixture, 0.01% (w/w) of fluorescent dye DilC_18_ was added in a small volume of methanol, and the volume of the mixture was adjusted to 5 mL with methanol. 500 µL of the lipid mixture was transferred into separate test tubes, and the solvent was removed under the stream of nitrogen gas.

### Reconstitution from CHAPS- or LDAO-micelles

Reconstitution from 1% CHAPS or 1% LDAO was performed on ExtractiGel detergent removing resin [19]. 3 mL of 50% slurry of ExtractiGel D detergent removing gel was packed into a 5 mL disposable polypropylene column (Thermo Scientific) and equilibrated with 3x5 mL PBS.

4 mL of 1% detergent was added to the test tube containing 10 mg of lipids and mixed well by pipetting up and down so that all the lipids were thoroughly solubilized. 800 µL of the detergent – lipid mixture was transferred into 1.5 mL Eppendorf tube containing 20 µg of the purified CB_2_ protein in 50 µL and 50 µL of the 1% CHAPS or LDAO, making the total volume of protein – detergent – lipid mixture 900 µL.

300 µL of protein – detergent – lipid mixture was loaded onto a pre-equilibrated ExtractiGel column. Flow-through was discarded, 300 µL of PBS buffer was loaded onto the column, and the eluate was discarded again. Another 1 ml of PBS was loaded on top of the column, and the first 800 µL of the elution fraction containing the liposomes were pooled and concentrated on a 30 kDa membrane filter (Apollo 20 ml concentrator) at 4°C. To this concentrated sample, 1 ml of PBS buffer was added, and sample concentrated to 100 µL. This cycle of diluting with PBS and concentrating 10-fold was repeated four more times.

Smaller preparations of proteoliposomes (typically, 10–20 µg of protein) were obtained on a pre-packed 0.5 ml detergent removal spin columns (Pierce, Rockford, IL).

### Preparation of Proteoliposomes for Solid-state NMR

Proteoliposomes for measurements of ligand binding by solid-state NMR were prepared as described elsewhere [19]. Briefly, 400 µg of CB_2_-130 supplemented with 1 mol % of the Alexa488-labeled CB_2_ was loaded onto 500 µL of Ni-NTA resin suspended at 50% (v/v) in the TD buffer supplemented with 10.0 µM CP-55,940 and incubated for 2 h at 4°C on a shaker. Upon binding of the receptor to Ni-NTA resin, the exchange of the unlabeled to labeled ligand was performed. After the immobilized receptor was washed on a column with 800 µL ×2 of the TD buffer, the ligand was exchanged by washing with the buffer (800 µL ×10) containing 9.08 µM of CP-55,940-*d*_6_ (a) with or (b) without addition of 90.8 µM of unlabeled CP-55,940. This exchange buffer also contained lipids (3.2 mg/mL POPC, 0.8 mg/mL POPS, and 1 mol% POPC-*d*_4_ supplemented with 0.4 µg/mL DilC_18_(5)) necessary for subsequent reconstitution steps. The protein was eluted from the resin with 200 µL ×5 of the same exchange buffer containing ligands and lipids, supplemented with 250 mM imidazole, at pH = 7.5. Due to the high lipophylicity of CP-55,940 both ligand and lipids were dissolved in detergent micelles; therefore, the molar ratio between the ligand and lipids was preserved through the exchange, elution, and the subsequent reconstitution steps [22], [48]. The quantification of ligand was performed by measuring the content of deuterated CP-55,940 by high resolution NMR. Quantification of the lipid was performed by measuring fluorescence of the labeled tracer (DilC_18_). Reconstitution of CB_2_ into proteoliposomes was performed by the rapid dilution method [19].

Proteoliposomes were precipitated by overnight centrifugation at 417,200×g at 4°C on the Optima TLX ultracentrifuge. Supernatant was discarded, and the proteoliposome pellet was re-dispersed in an equal amount (w/w) of de-ionized water. Each sample was then transferred into a 4-mm-o.d. zirconia MAS rotor with an insert made of Kel-F used to keep the sample centered within the rotor.

### Solid-State NMR Measurements

^2^H MAS NMR measurements of binding of CP-55,940-*d*_6_ to the receptor were conducted at sample temperature of 20 °C and 14.5 kHz MAS on a Bruker AV800 spectrometer operating at the resonance frequency of 122.83 MHz [19]. Interval time between 90° pulses was set to 250 ms in the acquisition to assure full recovery of the methyl signals of CP-55,940-*d*_6_ as well as the headgroup methylene signals of POPC-*d*_4_ used as a standard.

### Purification of *Gα_i1_* and *Gβ_1_γ_2_* Subunits

Myristoylated recombinant *Gα_i1_* was produced in *E. coli*, expressing both *Gα_i1_* and N-myristoyltransferase, following previously published procedure [29].

Heterodimeric *Gβ_1_γ_2_* were expressed in Sf9 cells [30] infected with baculoviruses encoding these subunits. P2 membranes were prepared, extracted with 1% sodium cholate, and *Gβ_1_γ_2_* purified essentially as described previously [30]. The purified proteins were stored in a solution of 10 mM MOPS, pH 7.5, 1 mM MgCl_2_, 100 mM NaCl with 8 mM CHAPS at -80°C.

### Activation of G Protein in an *in vitro* Coupled Assay

Activation of G proteins by the recombinant CB_2_ was performed according to the protocol previously reported [20] with some modifications.

Proteoliposomes containing reconstituted CB_2_ were diluted into ice-cold 10 mM MOPS buffer so that the final concentration of protein was 0.2–0.5 ng/µL. 10 µL of liposome emulsion containing 2 to 5 ng of the reconstituted CB_2_ was dispensed into the pre-siliconized glass tubes and mixed with cannabinoid ligand dispersed in 10 mM MOPS supplemented with 0.1% (w/v) BSA. Upon addition of a mixture of G_αi1_ (100 nM) and G_β1γ2_ (500 nM) the tubes were incubated on ice for 30 minutes. The reaction was started by addition of (final concentrations) MOPS buffer pH 7.5 (50 mM), EDTA (1 mM), MgCl_2_ (3 mM), GDP (4 µM), BSA (0.3% w/v), NaCl (100 mM), DTT (1 mM) and an appropriate amount of ^35^S-γ-GTP, and rapidly transferring the tube to the water bath set at 30°C. The total volume of the reaction was 50 µL. Incubation continued for 20 minutes and was terminated by the addition of 2 mL ice-cold stop solution TNMg (20 mM Tris-HCl pH 8.0, 100 mM NaCl, 25 mM MgCl_2_). The reaction was rapidly filtered through the nitrocellulose filters (Millipore). Filters were washed with 4 x 2 mL of cold TNMg buffer, dried, placed in the scintillation vials and counted upon addition of ScintiSafe Econo F scintillation liquid (Fisher).

### Activity Standard for G Protein Activation Assay

The *E. coli* membranes expressing CB_2_ were used as an activity standard since they lack endogenous G proteins and contain stable receptor accessible for interaction with G proteins. The levels of CB_2_ in *E. coli* CB_2_-130 membranes quantified by ligand binding assay as well as by semi-quantitative Western blot [5] are ∼3 ng of CB_2_ per 1 mg of membrane protein. The basal levels of activation of CB_2_ in *E. coli* membranes are negligible, while the response of the recombinant receptor to agonist stimulation is specific and ligand dose-dependent [20]. These membranes were provided in the amounts of 1–2 mg (3–6 ng of CB_2_) per assay. Reaction conditions were optimized to ensure that less than 30% of the available [^35^S] GTPγS was consumed.

### Analysis of the Residual Detergents in Liposomes by High Resolution ^1^H NMR

Content of CHS and residual detergents in proteoliposomes was determined by high-resolution solution-state ^1^H NMR as described earlier [19].

## Supporting Information

Figure S1 **Effect of detergents on rates of activation of G proteins by CB_2_-130.** Detergents at indicated concentrations were added to the reaction mix and measurements were performed shortly thereafter. Activity is presented as % of values obtained for the sample without addition of detergent. The results shown represent data ± S.D. (error bars) of duplicate measurements from representative experiments (n = 2).
(TIF)

Figure S2 **Effect of CHS in detergent micelles on activity of the purified CB_2_ (upon reconstitution into POPC/POPS/CHS liposomes).** Membranes of *E. coli* expressing fusion CB_2_-130 (2ug total protein) were used as an activity standard. Data ± S.D. of duplicate measurements from representative sample sets (n = 3) are shown.
(TIF)

Figure S3 **Effects of cannabinoid ligands in growth medium of** ***E. coli*** **BL21 (DE3) on expression levels and activity of fusion CB_2_-130.** Expression levels were determined by Western blot and the activity- by G protein activation assay. Duplicate measurements ± S.D. of activation rates of G proteins and expression levels of CB_2_ in a representative set of membranes are shown (n = 2).
(TIF)

Figure S4 **Pair-wise comparison of stabilizing effects of POPS and DOPS.** CB_2_ in DDM/CHAPS micelles containing either 0.1% POPC:POPS (50∶50) or POPC:DOPS (50∶50) was incubated either at 4°C or 37°C, was supplemented with either POPC:DOPS(50∶50) or POPC:POPS (50∶50) such that the lipid composition of all samples became: POPC:POPS:DOPS (50∶25:25), reconstituted into proteoliposomes, and functional activity determined by G protein activation assay. Shown are results ± S.D. of duplicate measurements from representative set of proteoliposomes (n = 3). Activity of CB_2_ incubated in micelles supplemented with POPC/POPS is set as 100%.
(TIF)

Figure S5 **^2^H MAS NMR spectra of CP-55,940-** ***d*** **_6_ in the CB_2_ proteoliposomes.** 1 mol% of POPC-*d*_4_ was used as a quantification standard with (green) or without (blue) 10-fold excess of unlabeled CP-55,940. The spectra were recorded on the Bruker AV800 spectrometer at 20 °C and MAS frequency of 14.5 kHz. Signal intensities of POPC-*d*_4_ were adjusted for comparison of the ligand signal. Asterisk denotes natural abundance ^2^H signal of lipids. Results are shown for proteoliposomes prepared from mixed micelles. A, containing 0.1% (w/v) CHS for stabilization of the protein structure, or B, no CHS. Intensity of α deuterium in POPC-*d*_4_ reflects the minute deviation from the magic angle adjusted for each set of experiments, but this does not affect the quantification of ligand binding as relative intensities of α and β signals are constant.
(TIF)

Figure S6 **Quantification of CP-55,940-** ***d_6_*** **binding to CB_2_ in POPC:POPS (40∶60) liposomes.** Quantities of CB_2_, phospholipids (POPC:POPS = 2∶3+1 mol% POPC-*d*_4_), and CP-55,940 ( deuterium labeled or unlabeled) in the proteoliposomes. (1) Proteoliposomes with labeled CP-55,940-*d*_6_: CB_2_ = 2.38×10^−9^ mol (105 mg); unlabeled phospholipids = 9.28×10^−6^ mol (7186 mg)); POPC-*d*_4_ = 9.28×10^−8^ mol; CP-55,940-*d*_6_ = 1.22×10^−8^ mol. The amounts of free CP-55,940-*d*_6_ in the lipid matrix and of bound CP-55,940-*d*_6_ in the binding pocket are estimated to be 9.82×10^−9^ and 2.38×10^−9^ mol, respectively, on the 1∶1 complex of CB_2_ and the ligand. (2) Proteoliposomes with labeled CP-55,940-*d*_6_ and 10-fold molar excess of unlabeled CP-55,940:CB_2_ = 2.22×10^−9^ mol (98 mg)); unlabeled phospholipids = 9.28×10^−6^ mol (7189 mg)); POPC-*d*_4_ = 9.28×10^−8^ mol; CP-55,940-*d*_6_ = 1.22×10^−8^ mol; CP-55,940 = 1.22×10^−7^ mol. The amounts of free and receptor-bound CP-55,940-*d*_6_ are estimated to be 1.20×10^−8^ and 2.22×10^−10^ mol, respectively. If 100% of CB_2_ is functional, increase of CP-55-*d*_6_ signal upon introduction of the excess of unlabeled CP-55,940 is estimated to be 22%. According to the G-protein activation test the batch of the purified receptor subjected to the ligand-exchange procedure exhibited ∼75% of functional activity. Therefore, the expected signal increase in the ^2^H MAS NMR is 17%. The observed 14% in signal intensity increase corresponds to ∼82% of recovery of ligand binding-competent CB_2_ in proteoliposome preparation.
(TIF)

Figure S7 **Long-term stability of CB_2_ in POPC/POPS/CHS proteoliposomes.** Proteoliposomes were stored either at 4°C or −80°C, samples withdrawn periodically and activity measured by the G protein activation assay. Activity is presented as % of the control (*E. coli* membranes expressing CB_2_-130). 6 ng of CB_2_ (either in *E. coli* membranes or in proteoliposomes) per reaction was used, and results are average of two measurements with S.D. indicated.
(TIF)

Table S1 **Efficiency of detergents in solubilization of recombinant CB_2_ from** ***E. coli*** **membranes.**
(PPTX)

Table S2 **Lipids for stabilization of CB_2_ in micelles.**
(DOCX)

## References

[1] Bockaert J, Pin JP (1999) Molecular tinkering of G protein-coupled receptors: an evolutionary success. Embo Journal 18: 1723–1729. 10202136 10.1093/emboj/18.7.1723 PMC1171258

[2] Sanchez C, de Ceballos ML, del Pulgar TG, Rueda D, Corbacho C, et al (2001) Inhibition of glioma growth in vivo by selective activation of the CB2 cannabinoid receptor. Cancer Research 61: 5784–5789. 11479216

[3] McKallip RJ, Lombard C, Fisher M, Martin BR, Ryu SH, et al (2002) Targeting CB2 cannabinoid receptors as a novel therapy to treat malignant lymphoblastic disease. Blood 100: 627–634. 12091357 10.1182/blood-2002-01-0098

[4] Pertwee RG (2002) Cannabinoids and multiple sclerosis. Pharmacology & Therapeutics 95: 165–174. 12182963 10.1016/s0163-7258(02)00255-3

[5] Yeliseev AA, Wong KK, Soubias O, Gawrisch K (2005) Expression of human peripheral cannabinoid receptor for structural studies. Protein Science 14: 2638–2653. 16195551 10.1110/ps.051550305 PMC2253291

[6] Yeliseev A, Vukoti K (2011) Expression of G-protein-coupled receptors. In: Robinson AS, editor. Production of membrane proteins. Weinheim: Wiley-VCH. 219–248.

[7] Sarramegna V, Muller I, Milon A, Talmont F (2006) Recombinant G protein-coupled receptors from expression to renaturation: a challenge towards structure. Cellular and Molecular Life Sciences 63: 1149–1164. 16568239 10.1007/s00018-005-5557-6 PMC11136100

[8] Grisshammer R (2009) Purification of recombinant G protein-coupled receptors. Guide to Protein Purification, Second Edition 463: 631–645. 10.1016/S0076-6879(09)63036-6 PMC4754789 19892196

[9] Lorch M, Batchelor R (2011) Stabilizing membrane proteins in detergents and lipid systems. In: Robinson AS, editor. Production of membrane proteins. Weinheim: Wiley-VCH. 361–390.

[10] Serrano-Vega MJ, Magnani F, Shibata Y, Tate CG (2008) Conformational thermostabilization of the beta 1-adrenergic receptor in a detergent-resistant form. Proceedings of the National Academy of Sciences of the United States of America 105: 877–882. 18192400 10.1073/pnas.0711253105 PMC2242685

[11] Shibata Y, White JF, Serrano-Vega MJ, Magnani F, Aloia AL, et al (2009) Thermostabilization of the neurotensin receptor NTS1. Journal of Molecular Biology 390: 262–277. 19422831 10.1016/j.jmb.2009.04.068 PMC2696590

[12] Jaakola VP, Griffith MT, Hanson MA, Cherezov V, Chien EYT, et al (2008) The 2.6 angstrom crystal structure of a human A(2A) adenosine receptor bound to an antagonist. Science 322: 1211–1217. 18832607 10.1126/science.1164772 PMC2586971

[13] Mancia F, Hendrickson WA (2007) Expression of recombinant G-protein coupled receptors for structural biology. Mol Biosyst 3: 723–734. 17882334 10.1039/B713558K

[14] Cherezov V, Rosenbaum DM, Hanson MA, Rasmussen SGF, Thian FS, et al (2007) High-resolution crystal structure of an engineered human beta(2)-adrenergic G protein-coupled receptor. Science 318: 1258–1265. 17962520 10.1126/science.1150577 PMC2583103

[15] Serrano-Vega MJ, Tate CG (2009) Transferability of thermostabilizing mutations between beta-adrenergic receptors. Molecular Membrane Biology 26: 385–396. 19883298 10.3109/09687680903208239

[16] Magnani F, Shibata Y, Serrano-Vega MJ, Tate CG (2008) Co-evolving stability and conformational homogeneity of the human adenosine A(2a) receptor. Proceedings of the National Academy of Sciences of the United States of America 105: 10744–10749. 18664584 10.1073/pnas.0804396105 PMC2504806

[17] Robertson N, Jazayeri A, Errey J, Baig A, Hurrell E, et al (2011) The properties of thermostabilised G protein-coupled receptors (StaRs) and their use in drug discovery. Neuropharmacology 60: 36–44. 20624408 10.1016/j.neuropharm.2010.07.001

[18] Lebon G, Warne T, Edwards PC, Bennett K, Langmead CJ, et al (2011) Agonist-bound adenosine A(2A) receptor structures reveal common features of GPCR activation. Nature 474: 521–U154. 21593763 10.1038/nature10136 PMC3146096

[19] Kimura T, Yeliseev AA, Vukoti K, Rhodes SD, Cheng K, et al (2012) Recombinant Cannabinoid Type 2 Receptor in Liposome Model Activates G Protein in Response to Anionic Lipid Constituents. Journal of Biological Chemistry 287: 4076–4087. 22134924 10.1074/jbc.M111.268425 PMC3281699

[20] Yeliseev A, Zoubak L, Gawrisch K (2007) Use of dual affinity tags for expression and purification of functional peripheral cannabinoid receptor. Protein Expression and Purification 53: 153–163. 17223358 10.1016/j.pep.2006.12.003 PMC1906715

[21] Gawrisch K, Soubias O (2008) Structure and dynamics of polyunsaturated hydrocarbon chains in lipid bilayers - significance for GPCR function. Chemistry and Physics of Lipids 153: 64–75. 18396152 10.1016/j.chemphyslip.2008.02.016 PMC2453513

[22] Kimura T, Cheng K, Rice KC, Gawrisch K (2009) Location, structure, and dynamics of the synthetic cannabinoid ligand CP-55,940 in lipid bilayers. Biophysical Journal 96: 4916–4924. 19527650 10.1016/j.bpj.2009.03.033 PMC2712040

[23] Zhang RD, Kim TK, Qiao ZH, Cai J, Pierce WM, et al (2007) Biochemical and mass spectrometric characterization of the human CB2 cannabinoid receptor expressed in Pichia pastoris - Importance of correct processing of the N-terminus. Protein Expression and Purification 55: 225–235. 17500008 10.1016/j.pep.2007.03.018

[24] Feng W, Cai H, Pierce WM, Song ZH (2002) Expression of CB2 cannabinoid receptor in Pichia pastoris. Protein Expression and Purification 26: 496–505. 12460775 10.1016/s1046-5928(02)00569-7

[25] Magnin T, Fiez-Vandal C, Potier N, Coquard A, Leray I, et al (2009) A novel, generic and effective method for the rapid purification of G protein-coupled receptors. Protein Expression and Purification 64: 1–7. 18835448 10.1016/j.pep.2008.09.007

[26] Link AJ, Skretas G, Strauch EM, Chari NS, Georgiou G (2008) Efficient production of membrane-integrated and detergent-soluble G protein-coupled receptors in Escherichia coli. Protein Science 17: 1857–1863. 18593817 10.1110/ps.035980.108 PMC2548370

[27] Filppula S, Yaddanapudi S, Mercier R, Xu W, Pavlopoulos S, et al (2004) Purification and mass spectroscopic analysis of human CB2 cannabinoid receptor expressed in the baculovirus system. Journal of Peptide Research 64: 225–236. 15613086 10.1111/j.1399-3011.2004.00188.x

[28] Krepkiy D, Wong K, Gawrisch K, Yeliseev A (2006) Bacterial expression of functional, biotinylated peripheral cannabinoid receptor CB2. Protein Expression and Purification 49: 60–70. 16621595 10.1016/j.pep.2006.03.002

[29] Mumby SM, Linder ME (1994) Myristoylation of G-protein alpha-subunits Heterotrimeric G Proteins. 237: 254–268. 10.1016/s0076-6879(94)37067-2 7935001

[30] Wildman DE, Tamir H, Leberer E, Northup JK, Dennis M (1993) Prenyl modification of guanine-nucleotide regulatory protein-gamma-2 subunits is not required for interaction with the transducin alpha-subunit or rhodopsin. Proceedings of the National Academy of Sciences of the United States of America 90: 794–798. 8430087 10.1073/pnas.90.3.794 PMC45756

[31] Harrison C, Traynor JR (2003) The ^S^35 GTP gamma-S binding assay: approaches and applications in pharmacology. Life Sciences 74: 489–508. 14609727 10.1016/j.lfs.2003.07.005

[32] Linder ME, Ewald DA, Miller RJ, Gilman AG (1990) Purification and characterization of G_o_-alpha and 3 types of G_i_-alpha after expression in *Escherichia coli* . Journal of Biological Chemistry 265: 8243–8251. 2159473

[33] Glass M, Northup JK (1999) Agonist selective regulation of g proteins by cannabinoid CB1 and CB2 receptors. Molecular Pharmacology 56: 1362–1369. 10570066 10.1124/mol.56.6.1362

[34] Kubota M, Tanaka T, Kohno T, Wakamatsu K (2009) GDP-GTP Exchange Processes of G alpha(i1) Protein are Accelerated/Decelerated Depending on the Type and the Concentration of Added Detergents. Journal of Biochemistry 146: 875–880. 19703944 10.1093/jb/mvp132

[35] Sarvazyan NA, Remmers AE, Neubig RR (1998) Determinants of G(i1)alpha and beta gamma binding - Measuring high affinity interactions in a lipid environment using flow cytometry. Journal of Biological Chemistry 273: 7934–7940. 9525890 10.1074/jbc.273.14.7934

[36] Sykora J, Bourova L, Hof M, Svoboda P (2009) The effect of detergents on trimeric G-protein activity in isolated plasma membranes from rat brain cortex: Correlation with studies of DPH and Laurdan fluorescence. Biochimica Et Biophysica Acta-Biomembranes 1788: 324–332. 10.1016/j.bbamem.2008.11.008 19071083

[37] Kobilka BK (2007) G protein coupled receptor structure and activation. Biochimica Et Biophysica Acta-Biomembranes 1768: 794–807. 10.1016/j.bbamem.2006.10.021 PMC1876727 17188232

[38] Grisshammer R, Averbeck P, Sohal AK (1999) Improved purification of a rat neurotensin receptor expressed in *Escherichia coli* . Biochemical Society Transactions 27: 899–903. 10830124 10.1042/bst0270899

[39] Ott D, Neldner Y, Cebe R, Dodevski I, Pluckthun A (2005) Engineering and functional immobilization of opioid receptors. Protein Engineering Design & Selection 18: 153–160. 10.1093/protein/gzi012 15790572

[40] Yao ZP, Kobilka B (2005) Using synthetic lipids to stabilize purified beta(2) adrenoceptor in detergent micelles. Analytical Biochemistry 343: 344–346. 16005425 10.1016/j.ab.2005.05.002

[41] Attrill H, Harding PJ, Smith E, Ross S, Watts A (2009) Improved yield of a ligand-binding GPCR expressed in *E. coli* for structural studies. Protein Expression and Purification 64: 32–38. 18976711 10.1016/j.pep.2008.10.001

[42] Niu SL, Mitchell DC, Lim SY, Wen ZM, Kim HY, et al (2004) Reduced G protein-coupled signaling efficiency in retinal rod outer segments in response to n-3 fatty acid deficiency. J Biol Chem 279: 31098–31104. 15145938 10.1074/jbc.M404376200

[43] Inagaki S, Ghirlando R, White JF, Gvozdenovic-Jeremic J, Northup JK, et al (2012) Modulation of the interaction between neurotensin receptor NTS1 and Gq protein by lipid. J Mol Biol 417: 95–111. 22306739 10.1016/j.jmb.2012.01.023 PMC3294418

[44] O'Malley MA, Helgeson ME, Wagner NJ, Robinson AS (2011) The Morphology and Composition of Cholesterol-Rich Micellar Nanostructures Determine Transmembrane Protein (GPCR) Activity. Biophysical Journal 100: L11–L13. 21244820 10.1016/j.bpj.2010.12.3698 PMC3021673

[45] Lin SS, Gether U, Kobilka BK (1996) Ligand stabilization of the beta(2) adrenergic receptor: Effect of DTT on receptor conformation monitored by circular dichroism and fluorescence spectroscopy. Biochemistry 35: 14445–14451. 8931540 10.1021/bi961619+

[46] Pello OM, Martinez-Munoz L, Parrillas V, Serrano A, Rodriguez-Frade JM, et al (2008) Ligand stabilization of CXCR4/delta-opioid receptor heterodimers reveals a mechanism for immune response regulation. European Journal of Immunology 38: 537–549. 18200497 10.1002/eji.200737630

[47] Ratnala VRP (2006) New tools for G-protein coupled receptor (GPCR) drug discovery: combination of baculoviral expression system and solid state NMR. Biotechnology Letters 28: 767–778. 16786240 10.1007/s10529-006-9005-y

[48] Thomas BF, Compton DR, Martin BR (1990) Characterization of the lipophilicity of natural and synthetic analogs of delta-9-tetrahydrocannabinol and its relationship to pharmacological potency. Journal of Pharmacology and Experimental Therapeutics 255: 624–630. 2173751

