# Supplementary figures and images for: Stabilization of Functional Recombinant Cannabinoid Receptor CB2 in Detergent Micelles and Lipid Bilayers
Source: PLoS One. 2012 Oct 3;7(10):e46290. doi: 10.1371/journal.pone.0046290 (PMC3463599; doi:10.1371/journal.pone.0046290)

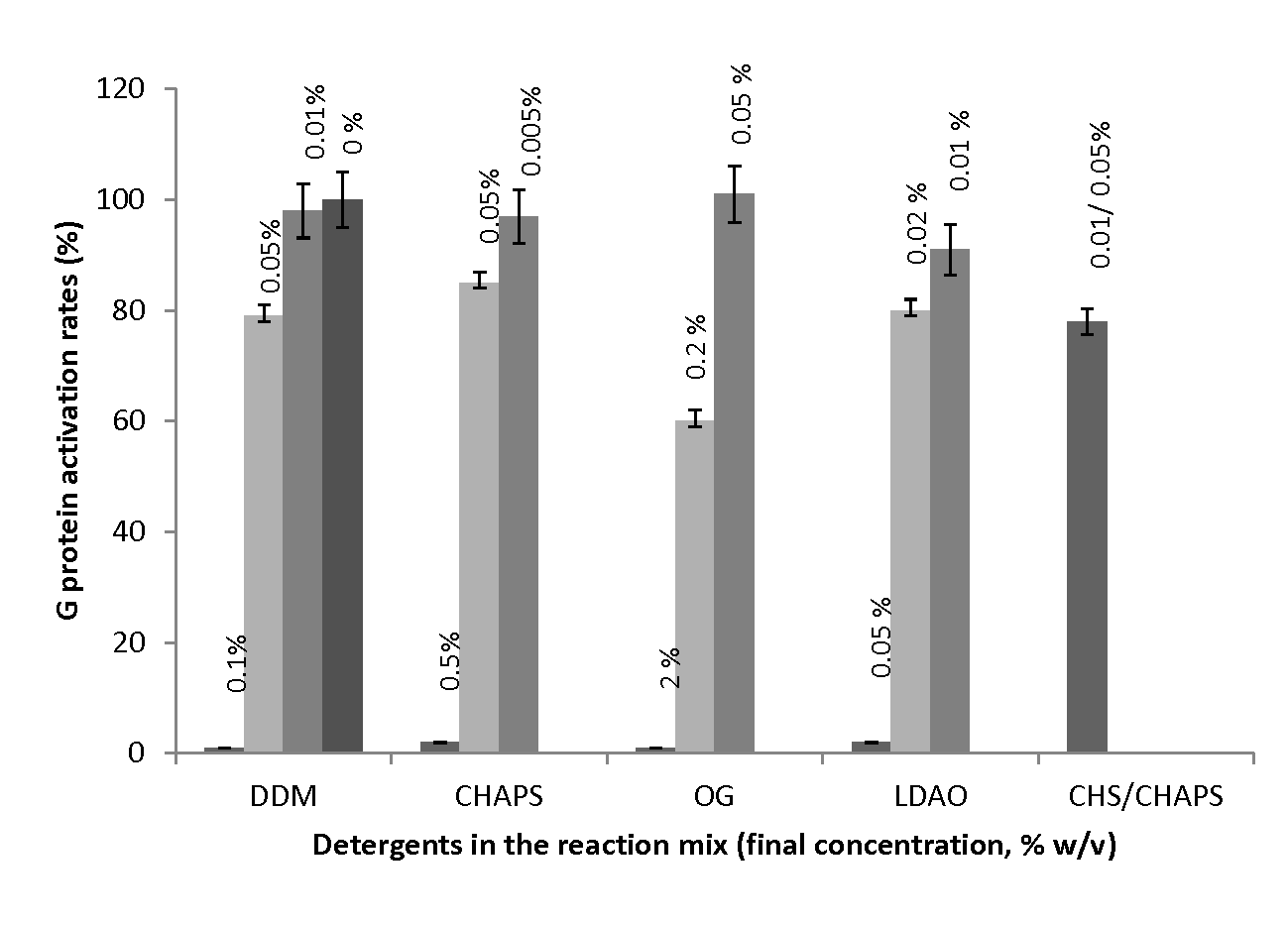

Supplement: Figure S1 — Effect of detergents on rates of activation of G proteins by CB2-130. Detergents at indicated concentrations were added to the reaction mix and measurements were performed shortly thereafter. Activity is presented as % of values obtained for the sample without addition of detergent. The results shown represent data ± S.D. (error bars) of duplicate measurements from representative experiments (n = 2). (TIF) [file pone.0046290.s001.tif]

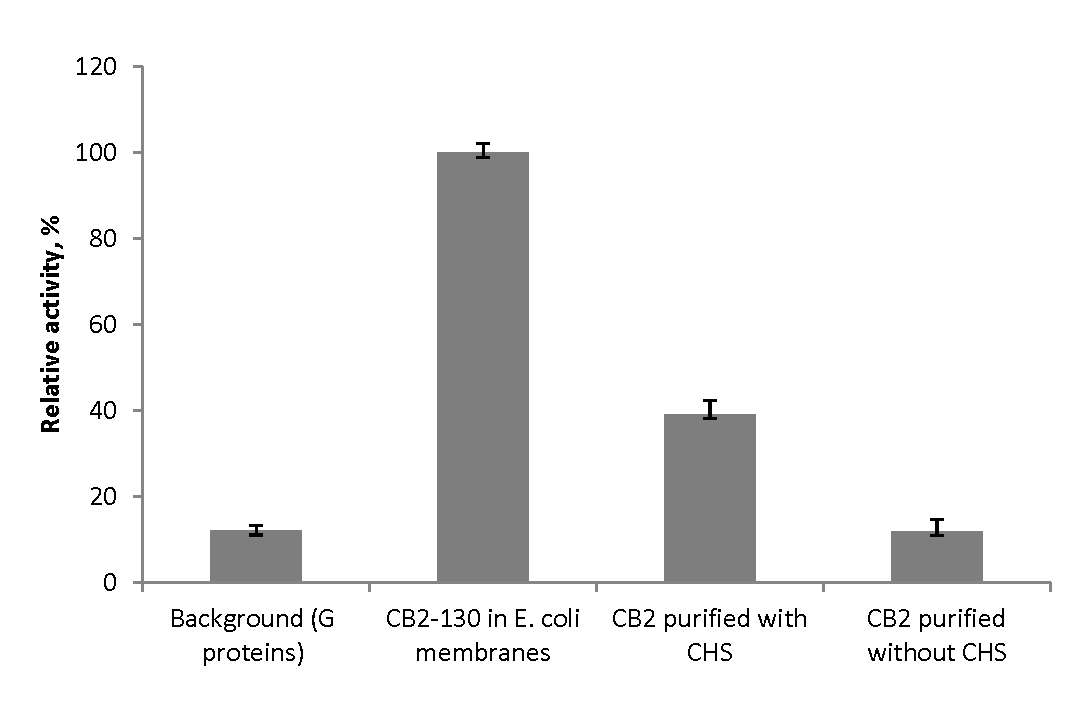

Supplement: Figure S2 — Effect of CHS in detergent micelles on activity of the purified CB2 (upon reconstitution into POPC/POPS/CHS liposomes). Membranes of E. coli expressing fusion CB2-130 (2ug total protein) were used as an activity standard. Data ± S.D. of duplicate measurements from representative sample sets (n = 3) are shown. (TIF) [file pone.0046290.s002.tif]

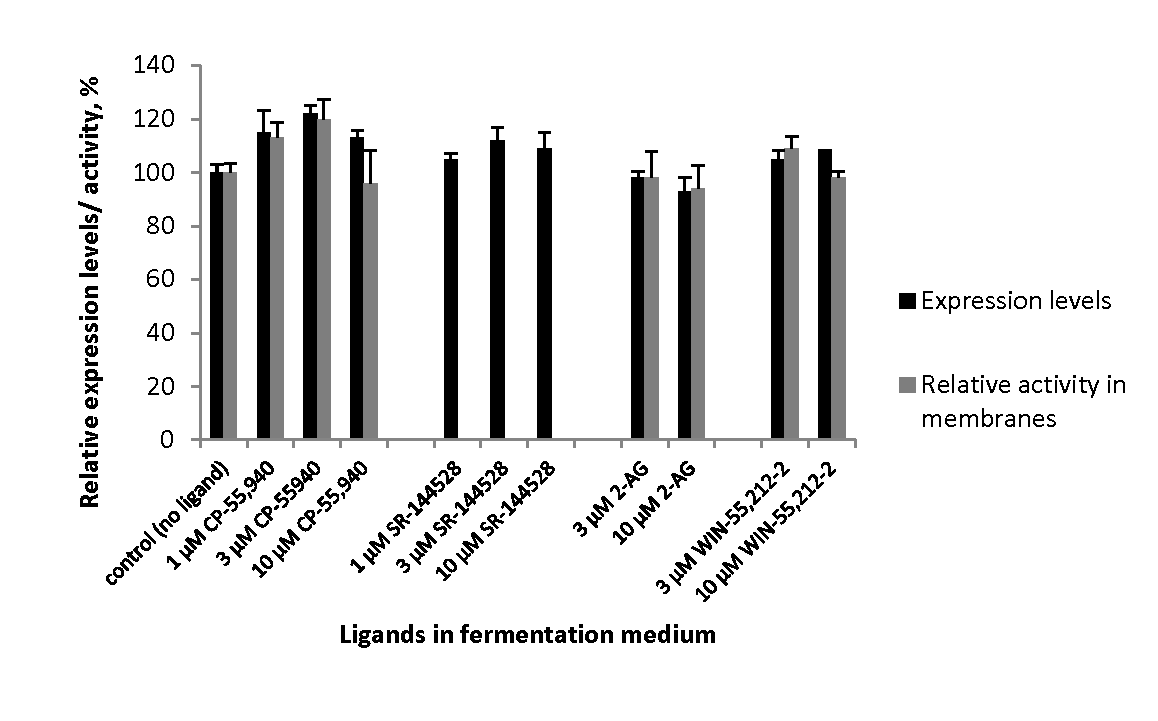

Supplement: Figure S3 — Effects of cannabinoid ligands in growth medium of E. coli BL21 (DE3) on expression levels and activity of fusion CB2-130. Expression levels were determined by Western blot and the activity- by G protein activation assay. Duplicate measurements ± S.D. of activation rates of G proteins and expression levels of CB2 in a representative set of membranes are shown (n = 2). (TIF) [file pone.0046290.s003.tif]

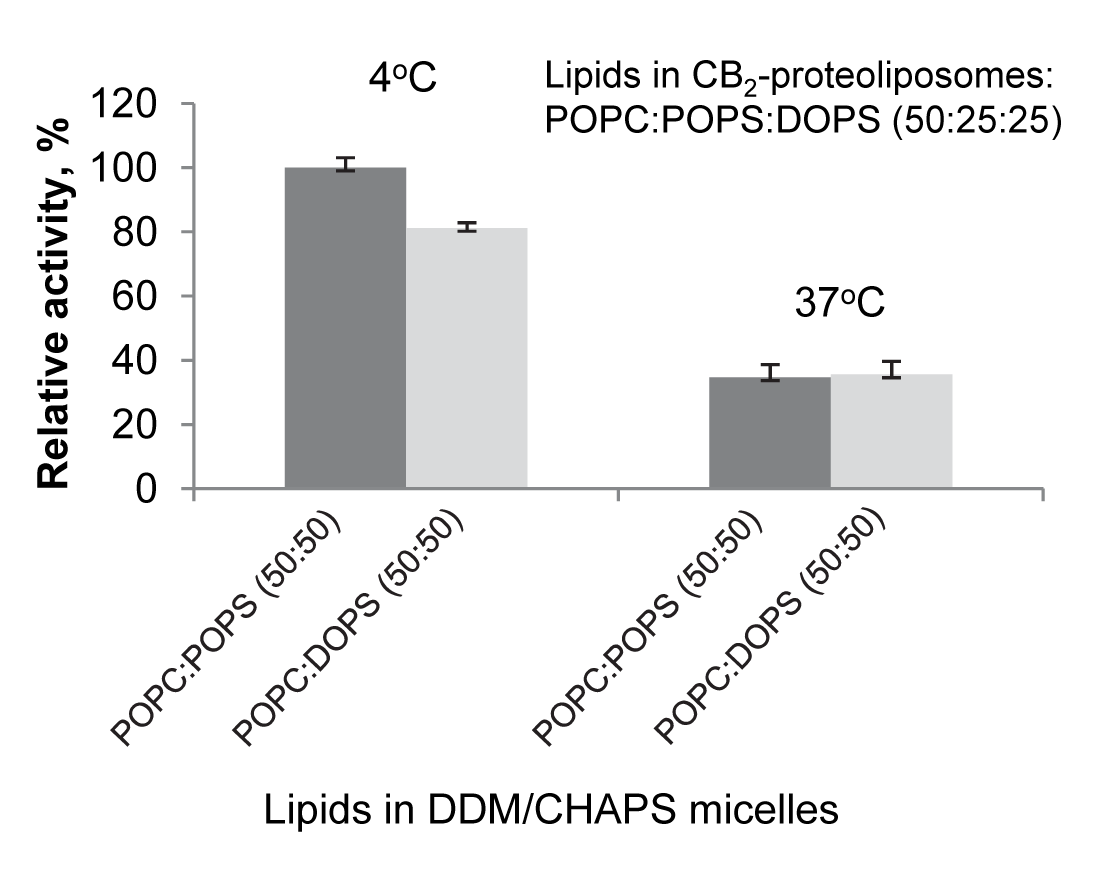

Supplement: Figure S4 — Pair-wise comparison of stabilizing effects of POPS and DOPS. CB2 in DDM/CHAPS micelles containing either 0.1% POPC:POPS (50∶50) or POPC:DOPS (50∶50) was incubated either at 4°C or 37°C, was supplemented with either POPC:DOPS(50∶50) or POPC:POPS (50∶50) such that the lipid composition of all samples became: POPC:POPS:DOPS (50∶25:25), reconstituted into proteoliposomes, and functional activity determined by G protein activation assay. Shown are results ± S.D. of duplicate measurements from representative set of proteoliposomes (n = 3). Activity of CB2 incubated in micelles supplemented with POPC/POPS is set as 100%. (TIF) [file pone.0046290.s004.tif]

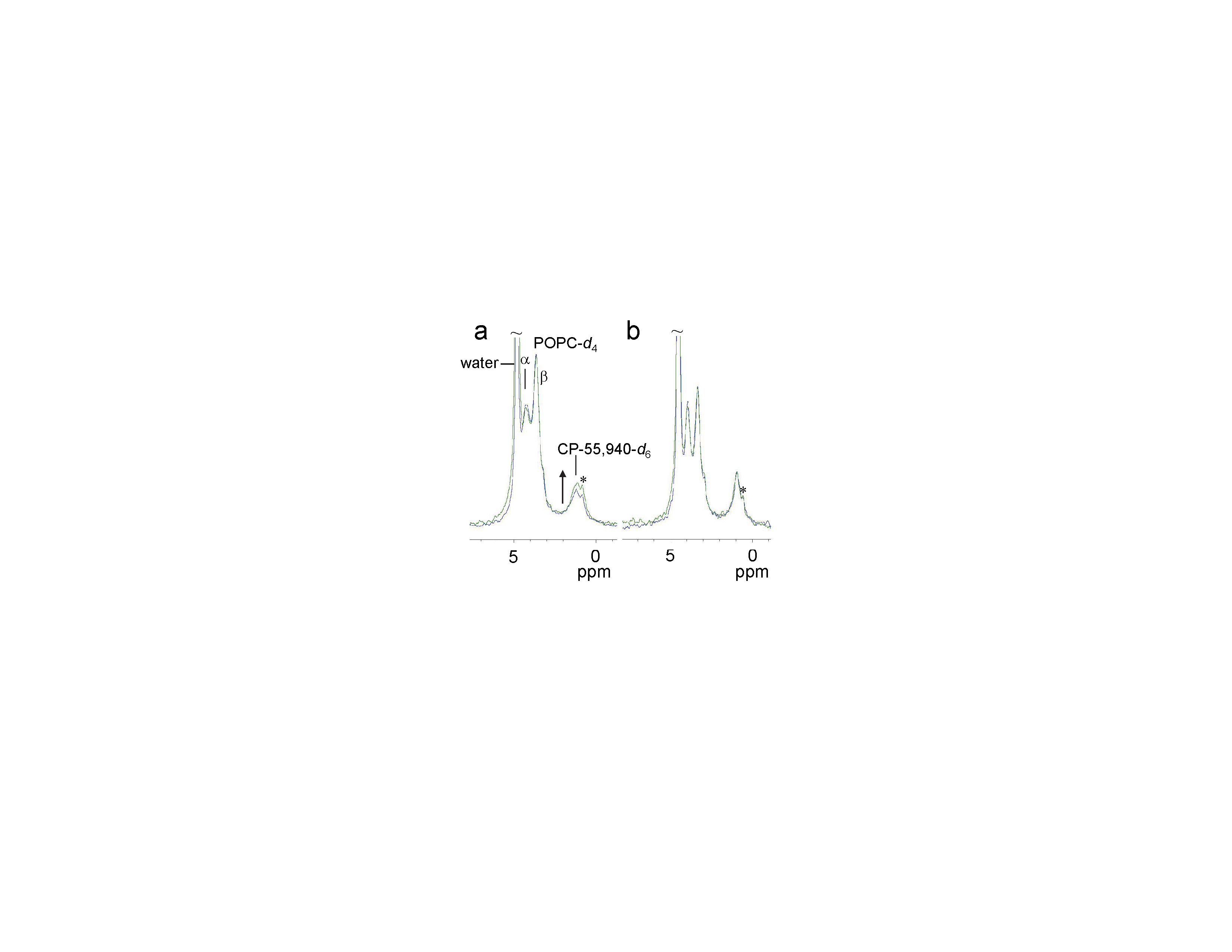

Supplement: Figure S5 — 2H MAS NMR spectra of CP-55,940- d 6 in the CB2 proteoliposomes. 1 mol% of POPC-d 4 was used as a quantification standard with (green) or without (blue) 10-fold excess of unlabeled CP-55,940. The spectra were recorded on the Bruker AV800 spectrometer at 20 °C and MAS frequency of 14.5 kHz. Signal intensities of POPC-d 4 were adjusted for comparison of the ligand signal. Asterisk denotes natural abundance 2H signal of lipids. Results are shown for proteoliposomes prepared from mixed micelles. A, containing 0.1% (w/v) CHS for stabilization of the protein structure, or B, no CHS. Intensity of α deuterium in POPC-d 4 reflects the minute deviation from the magic angle adjusted for each set of experiments, but this does not affect the quantification of ligand binding as relative intensities of α and β signals are constant. (TIF) [file pone.0046290.s005.tif]

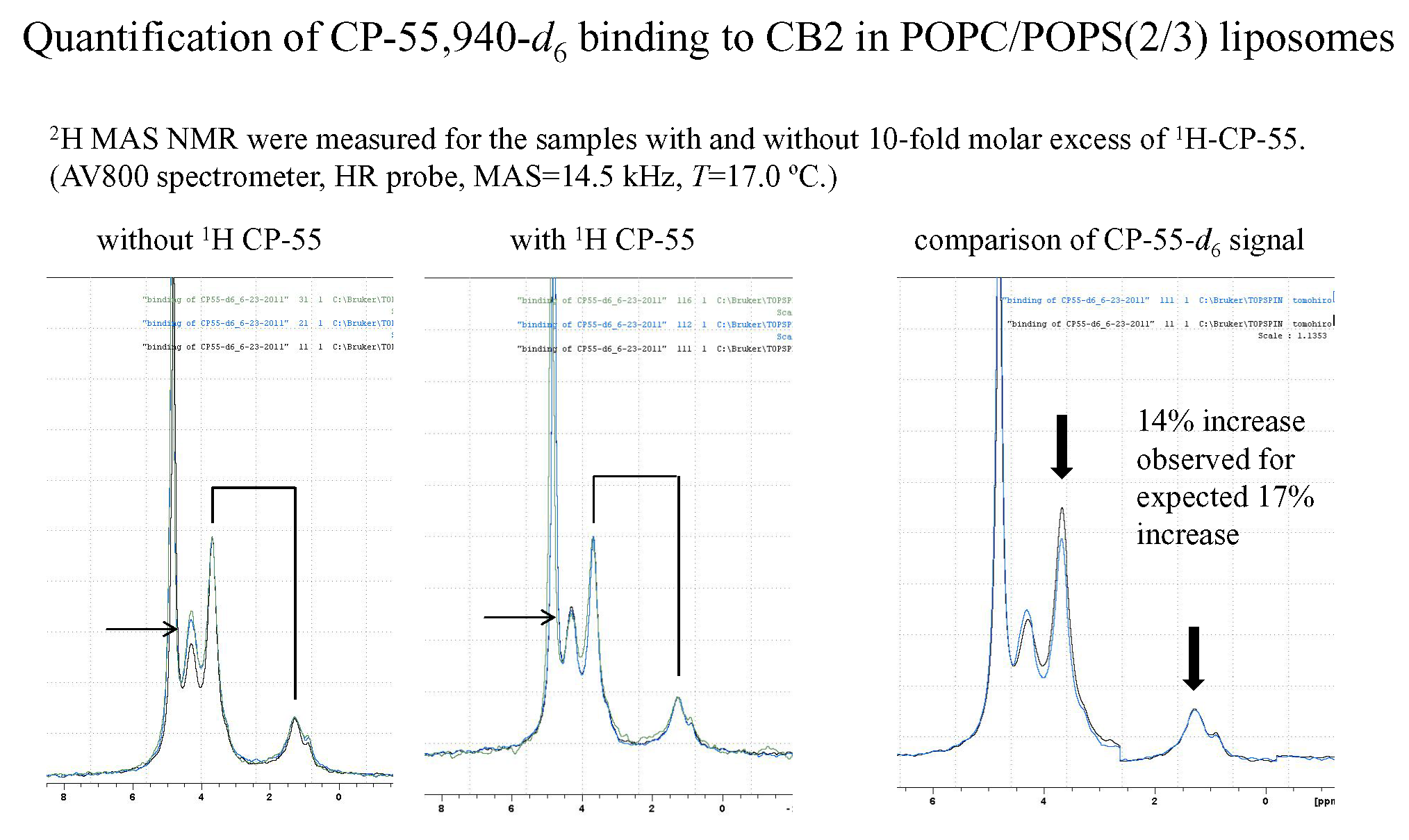

Supplement: Figure S6 — Quantification of CP-55,940- d6 binding to CB2 in POPC:POPS (40∶60) liposomes. Quantities of CB2, phospholipids (POPC:POPS = 2∶3+1 mol% POPC-d 4), and CP-55,940 ( deuterium labeled or unlabeled) in the proteoliposomes. (1) Proteoliposomes with labeled CP-55,940-d 6: CB2 = 2.38×10^−9^ mol (105 mg); unlabeled phospholipids = 9.28×10^−6^ mol (7186 mg)); POPC-d 4 = 9.28×10^−8^ mol; CP-55,940-d 6 = 1.22×10^−8^ mol. The amounts of free CP-55,940-d 6 in the lipid matrix and of bound CP-55,940-d 6 in the binding pocket are estimated to be 9.82×10^−9^ and 2.38×10^−9^ mol, respectively, on the 1∶1 complex of CB2 and the ligand. (2) Proteoliposomes with labeled CP-55,940-d 6 and 10-fold molar excess of unlabeled CP-55,940:CB2 = 2.22×10^−9^ mol (98 mg)); unlabeled phospholipids = 9.28×10^−6^ mol (7189 mg)); POPC-d 4 = 9.28×10^−8^ mol; CP-55,940-d 6 = 1.22×10^−8^ mol; CP-55,940 = 1.22×10^−7^ mol. The amounts of free and receptor-bound CP-55,940-d 6 are estimated to be 1.20×10^−8^ and 2.22×10^−10^ mol, respectively. If 100% of CB2 is functional, increase of CP-55-d 6 signal upon introduction of the excess of unlabeled CP-55,940 is estimated to be 22%. According to the G-protein activation test the batch of the purified receptor subjected to the ligand-exchange procedure exhibited ∼75% of functional activity. Therefore, the expected signal increase in the 2H MAS NMR is 17%. The observed 14% in signal intensity increase corresponds to ∼82% of recovery of ligand binding-competent CB2 in proteoliposome preparation. (TIF) [file pone.0046290.s006.tif]

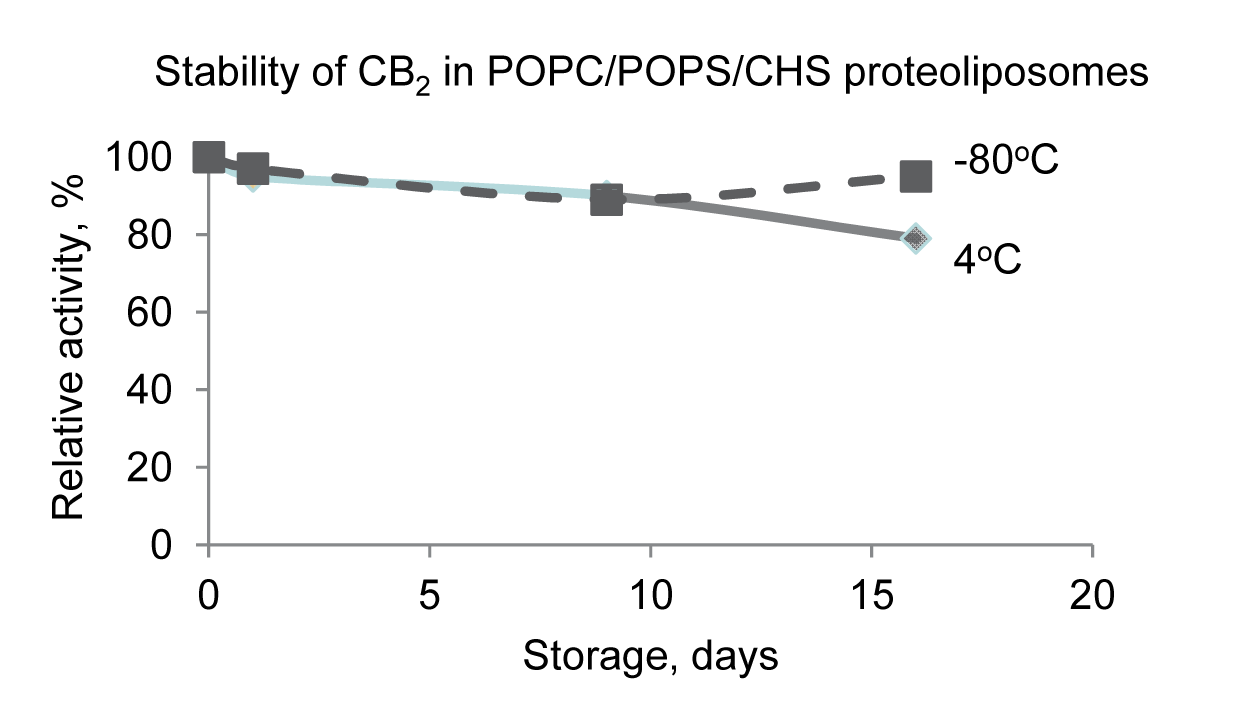

Supplement: Figure S7 — Long-term stability of CB2 in POPC/POPS/CHS proteoliposomes. Proteoliposomes were stored either at 4°C or −80°C, samples withdrawn periodically and activity measured by the G protein activation assay. Activity is presented as % of the control (E. coli membranes expressing CB2-130). 6 ng of CB2 (either in E. coli membranes or in proteoliposomes) per reaction was used, and results are average of two measurements with S.D. indicated. (TIF) [file pone.0046290.s007.tif]
