# Supplementary material for: Stabilization of Functional Recombinant Cannabinoid Receptor CB2 in Detergent Micelles and Lipid Bilayers
Source: PLoS One. 2012 Oct 3;7(10):e46290. doi: 10.1371/journal.pone.0046290 (PMC3463599; doi:10.1371/journal.pone.0046290)
Supplement: Table S1 — Efficiency of detergents in solubilization of recombinant CB2 from E. coli membranes. (PPTX) [file pone.0046290.s008.pptx]

## Slide 1
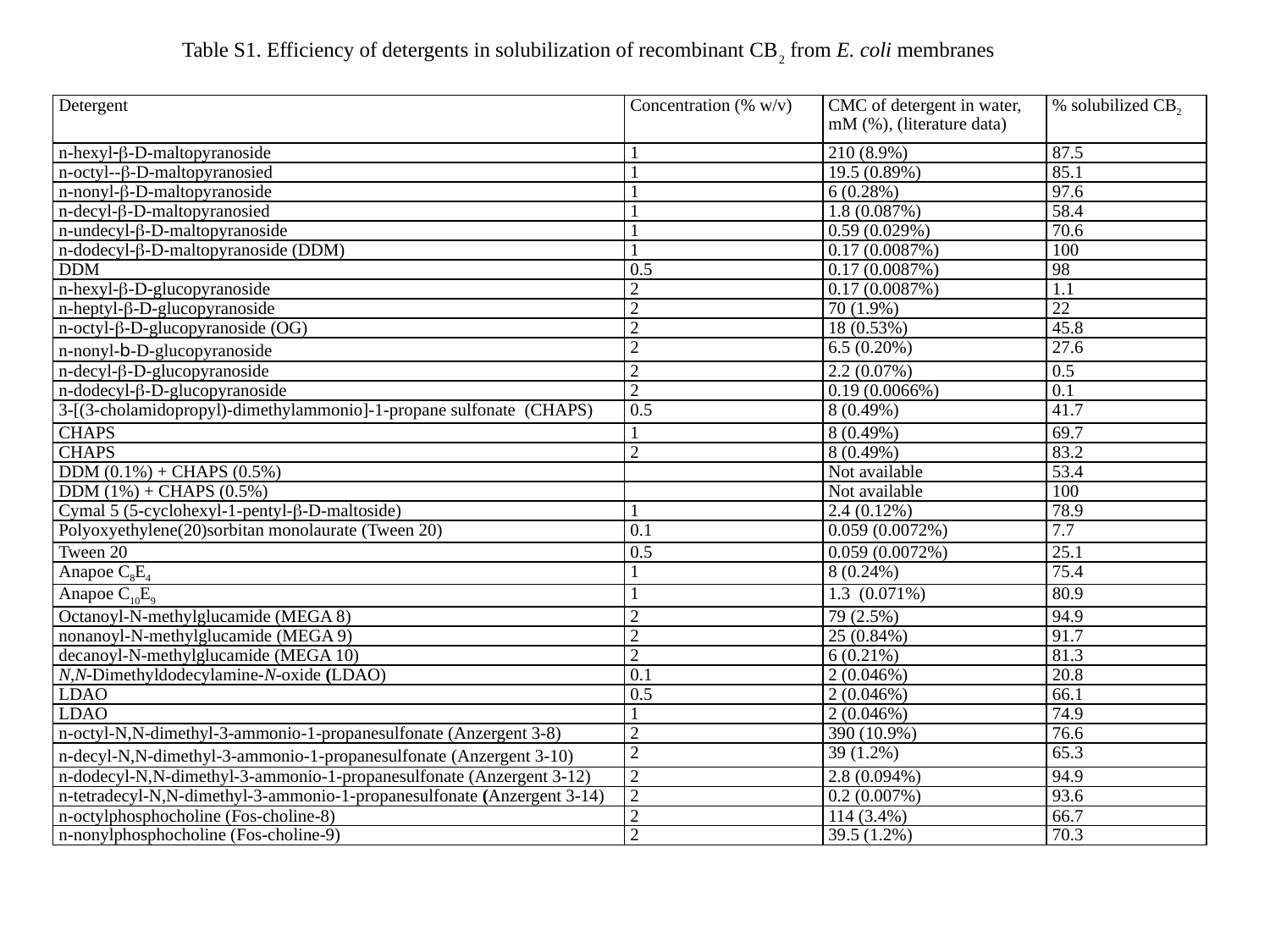

Table S1. Efficiency of detergents in solubilization of recombinant CB2 from E. coli membranes
| Detergent | Concentration (% w/v) | CMC of detergent in water, mM (%), (literature data) | % solubilized CB2 |
| --- | --- | --- | --- |
| n-hexyl-b-D-maltopyranoside | 1 | 210 (8.9%) | 87.5 |
| n-octyl--b-D-maltopyranosied | 1 | 19.5 (0.89%) | 85.1 |
| n-nonyl-b-D-maltopyranoside | 1 | 6 (0.28%) | 97.6 |
| n-decyl-b-D-maltopyranosied | 1 | 1.8 (0.087%) | 58.4 |
| n-undecyl-b-D-maltopyranoside | 1 | 0.59 (0.029%) | 70.6 |
| n-dodecyl-b-D-maltopyranoside (DDM) | 1 | 0.17 (0.0087%) | 100 |
| DDM | 0.5 | 0.17 (0.0087%) | 98 |
| n-hexyl-b-D-glucopyranoside | 2 | 0.17 (0.0087%) | 1.1 |
| n-heptyl-b-D-glucopyranoside | 2 | 70 (1.9%) | 22 |
| n-octyl-b-D-glucopyranoside (OG) | 2 | 18 (0.53%) | 45.8 |
| n-nonyl-b-D-glucopyranoside | 2 | 6.5 (0.20%) | 27.6 |
| n-decyl-b-D-glucopyranoside | 2 | 2.2 (0.07%) | 0.5 |
| n-dodecyl-b-D-glucopyranoside | 2 | 0.19 (0.0066%) | 0.1 |
| 3-[(3-cholamidopropyl)-dimethylammonio]-1-propane sulfonate (CHAPS) | 0.5 | 8 (0.49%) | 41.7 |
| CHAPS | 1 | 8 (0.49%) | 69.7 |
| CHAPS | 2 | 8 (0.49%) | 83.2 |
| DDM (0.1%) + CHAPS (0.5%) | | Not available | 53.4 |
| DDM (1%) + CHAPS (0.5%) | | Not available | 100 |
| Cymal 5 (5-cyclohexyl-1-pentyl-b-D-maltoside) | 1 | 2.4 (0.12%) | 78.9 |
| Polyoxyethylene(20)sorbitan monolaurate (Tween 20) | 0.1 | 0.059 (0.0072%) | 7.7 |
| Tween 20 | 0.5 | 0.059 (0.0072%) | 25.1 |
| Anapoe C8E4 | 1 | 8 (0.24%) | 75.4 |
| Anapoe C10E9 | 1 | 1.3 (0.071%) | 80.9 |
| Octanoyl-N-methylglucamide (MEGA 8) | 2 | 79 (2.5%) | 94.9 |
| nonanoyl-N-methylglucamide (MEGA 9) | 2 | 25 (0.84%) | 91.7 |
| decanoyl-N-methylglucamide (MEGA 10) | 2 | 6 (0.21%) | 81.3 |
| N,N-Dimethyldodecylamine-N-oxide (LDAO) | 0.1 | 2 (0.046%) | 20.8 |
| LDAO | 0.5 | 2 (0.046%) | 66.1 |
| LDAO | 1 | 2 (0.046%) | 74.9 |
| n-octyl-N,N-dimethyl-3-ammonio-1-propanesulfonate (Anzergent 3-8) | 2 | 390 (10.9%) | 76.6 |
| n-decyl-N,N-dimethyl-3-ammonio-1-propanesulfonate (Anzergent 3-10) | 2 | 39 (1.2%) | 65.3 |
| n-dodecyl-N,N-dimethyl-3-ammonio-1-propanesulfonate (Anzergent 3-12) | 2 | 2.8 (0.094%) | 94.9 |
| n-tetradecyl-N,N-dimethyl-3-ammonio-1-propanesulfonate (Anzergent 3-14) | 2 | 0.2 (0.007%) | 93.6 |
| n-octylphosphocholine (Fos-choline-8) | 2 | 114 (3.4%) | 66.7 |
| n-nonylphosphocholine (Fos-choline-9) | 2 | 39.5 (1.2%) | 70.3 |
