# Supplementary material for: Stabilization of Functional Recombinant Cannabinoid Receptor CB2 in Detergent Micelles and Lipid Bilayers
Source: PLoS One. 2012 Oct 3;7(10):e46290. doi: 10.1371/journal.pone.0046290 (PMC3463599; doi:10.1371/journal.pone.0046290)
Supplement: Table S2 — Lipids for stabilization of CB2 in micelles. (DOCX) [file pone.0046290.s009.docx]

Table S2. Lipids for stabilization of CB_2_ in micelles

| **Lipid** | **Structure** | **Reported phase transition temperature, °C^1^** |
| --- | --- | --- |
| POPC | 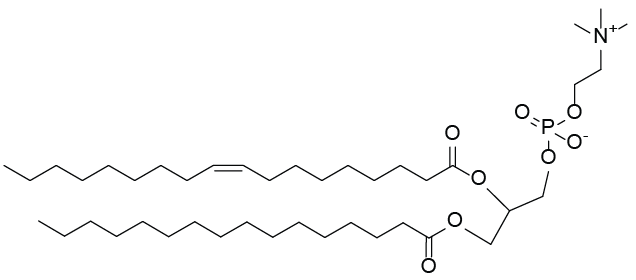 | -2 |
| DMPC | 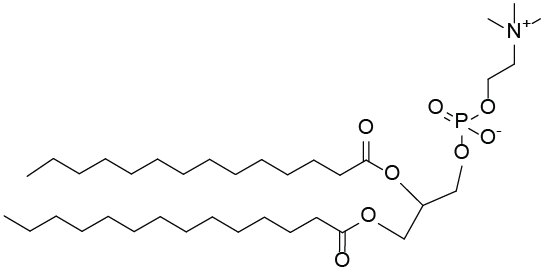 | 23 |
| SOPC | 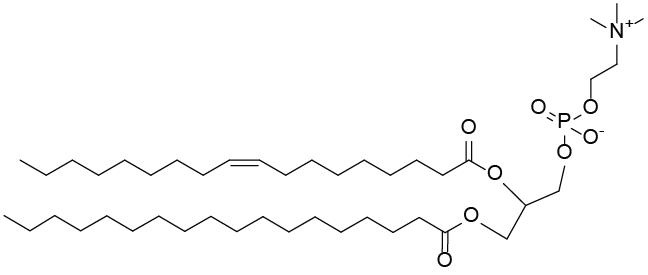 | 6 |
| POPS | 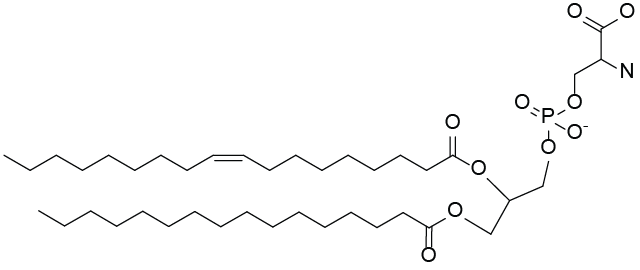 | 14 |
| DOPS | 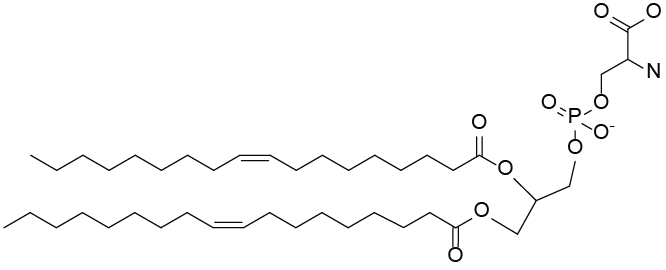 | 11 |
| POPG | 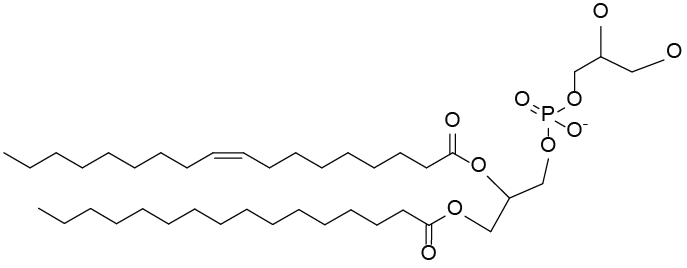 | -2 |
| DOPE | 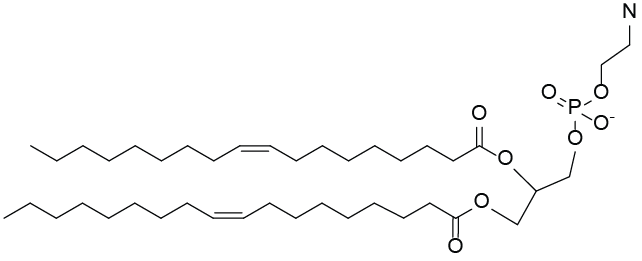 | -16 |
| Cholesterol hemisuccinate  (CHS) | 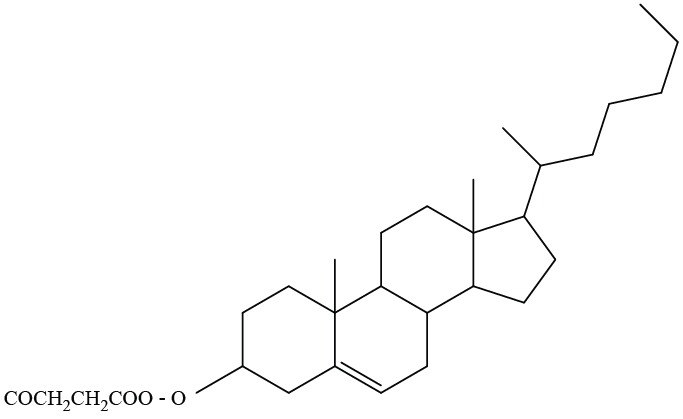 | nd |
| *E. coli* Cardiolipin  (Predominant species) | 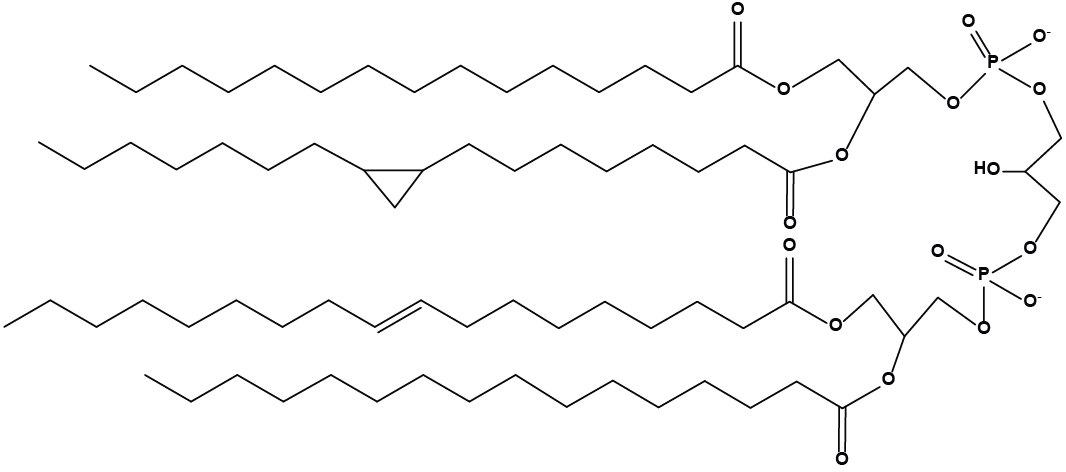 | nd |

^1^ (Avanti polar lipids)
